# Impaired Pre‐Motor Circuit Activity and Movement in a *Drosophila* Model of *KCNMA1*‐Linked Dyskinesia

**DOI:** 10.1002/mds.28479

**Published:** 2021-01-15

**Authors:** Patrick Kratschmer, Simon A. Lowe, Edgar Buhl, Ko‐Fan Chen, Dimitri M. Kullmann, Alan Pittman, James J.L. Hodge, James E.C. Jepson

**Affiliations:** ^1^ Department of Clinical and Experimental Epilepsy UCL Queen Square Institute of Neurology London United Kingdom; ^2^ School of Physiology, Pharmacology and Neuroscience University of Bristol Bristol United Kingdom; ^3^ Department of Genetics and Genome Biology University of Leicester Leicester United Kingdom; ^4^ Genetics Research Centre, St George's University of London London United Kingdom

**Keywords:** *Drosophila*, BK channel, *slowpoke*, paroxysmal dyskinesia, central pattern generator, pre‐motor circuit, locomotion

## Abstract

**Background:**

Paroxysmal dyskinesias (PxDs) are characterized by involuntary movements and altered pre‐motor circuit activity. Causative mutations provide a means to understand the molecular basis of PxDs. Yet in many cases, animal models harboring corresponding mutations are lacking. Here we utilize the fruit fly, *Drosophila*, to study a PxD linked to a gain‐of‐function (GOF) mutation in the *KCNMA1*/hSlo1 BK potassium channel.

**Objectives:**

We aimed to recreate the equivalent BK (big potassium) channel mutation in *Drosophila*. We sought to determine how this mutation altered action potentials (APs) and synaptic release in vivo; to test whether this mutation disrupted pre‐motor circuit function and locomotion; and to define neural circuits involved in locomotor disruption.

**Methods:**

We generated a knock‐in *Drosophila* model using homologous recombination. We used electrophysiological recordings and calcium‐imaging to assess AP shape, neurotransmission, and the activity of the larval pre‐motor central pattern generator (CPG). We used video‐tracking and automated systems to measure movement, and developed a genetic method to limit BK channel expression to defined circuits.

**Results:**

Neuronal APs exhibited reduced width and an enhanced afterhyperpolarization in the PxD model. We identified calcium‐dependent reductions in neurotransmitter release, dysfunction of the CPG, and corresponding alterations in movement, in model larvae. Finally, we observed aberrant locomotion and dyskinesia‐like movements in adult model flies, and partially mapped the impact of GOF BK channels on movement to cholinergic neurons.

**Conclusion:**

Our model supports a link between BK channel GOF and hyperkinetic movements, and provides a platform to dissect the mechanistic basis of PxDs. © 2021 The Authors. *Movement Disorders* published by Wiley Periodicals LLC on behalf of International Parkinson and Movement Disorder Society

Paroxysmal dyskinesias (PxDs) are characterized by intermittent attacks of dystonic, choreiform, and/or ballistic movements.^1^ Distinct forms of PxD can be clinically differentiated based on the triggers for attacks. These include paroxysmal kinesigenic dyskinesia (triggered by sudden movement), paroxysmal exercise‐induced dyskinesia, and paroxysmal non‐kinesigenic dyskinesia (PNKD; often triggered by alcohol, caffeine, stress, and fatigue).^2^ Here we focus on a PxD subtype whose neuropathological basis has not been investigated in an animal model: type‐3 PNKD (PNKD3; OMIM #609446).

PNKD3 is caused by autosomal dominant or de novo mutations in the *KCNMA1* locus, which encodes the pore‐forming hSlo1 α‐subunit of the calcium (Ca^2+^)‐activated BK (big potassium) channel.^3^, ^4^, ^5^, ^6^, ^7^, ^8^ BK channels modulate neuronal excitability and action potential (AP) firing rate by contributing to the repolarization and afterhyperpolarization (AHP) phases of APs.^9^, ^10^, ^11^ BK channels also limit neurotransmitter release at a variety of nerve terminals by driving inactivation of presynaptic voltage‐gated Ca^2+^ channels.^11^ The first mutation linked to PNKD3 was a dominant missense mutation (1301A → G) in exon 10 of *KCNMA1*. This mutation results in the replacement of a negatively charged aspartic acid residue with a neutral glycine (D434G) in hSlo1,^4^ and is associated with paroxysmal dystonic and choreiform movements of the mouth, tongue, and extremities. Electrophysiological analyses performed in non‐excitable cells indicate that D434G acts as a gain‐of‐function (GOF) mutation by increasing BK channel Ca^2+^ sensitivity, accelerating activation, and decelerating deactivation.^3^, ^4^, ^5^, ^12^ However, the impact of the D434G mutation on AP shape and neurotransmission in vivo has remained unclear. More fundamentally, this mutation has only been identified in a single multi‐generation family,^4^ and there are currently no animal models of D434G or any other BK channel mutation linked to PNKD3. Demonstrating that equivalent mutations disrupt movement in non‐human models would strongly support a causative link between BK channel GOF and involuntary movements, and provide a platform for mechanistic studies.

We therefore generated a knock‐in *Drosophila* model of PNKD3. Utilizing this model, we provide in vivo evidence that the equivalent mutation to D434G in *Drosophila* alters AP waveforms and neurotransmitter release, disrupts structured activity of pre‐motor circuits, and perturbs coordinated movement in both larval and adult *Drosophila*. Furthermore, we partially map the impact of GOF BK channels on adult movement to cholinergic neurons. Our results provide independent support for the genetic linkage between hSlo1 D434G and PxD, and suggest a critical and conserved role for BK channels in the regulation of movement across distantly related bilateral species.

## 1 Materials and Methods

See Supplemental Information for details of Materials and Methods.

## 2 Results

### 2.1 Generation of a *Drosophila* Model of PNKD3

**FIG. 1:**
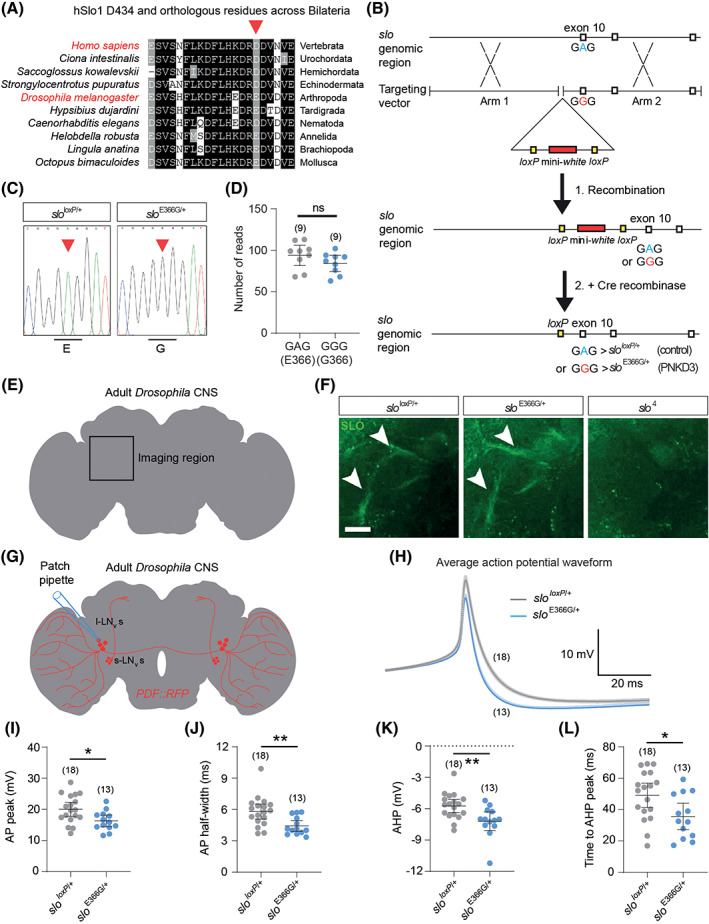
(**A**) Alignment of residues surrounding hSlo1 D434 (arrow) with orthologous BK α‐subunits from bilateral species spanning >540 million years of evolutionary divergence. (**B**) Schematic illustrating the procedure to generate the *slo* ^E366G^ and *slo* ^*loxP*^ alleles via ends‐out homologous recombination. The region surrounding exon 10 of the *slo* locus, which encodes the E366 residue, and corresponding targeting arms to induce homologous recombination, are shown. (**C**) Sanger sequence verification of the presence or absence of the A > G mutation in *slo* ^E366G/+^ and *slo* ^*loxP*/+^ flies via allele‐specific polymerase chain reaction (PCR). (**D**) RNAseq‐based quantification of *slo* ^+^ (GAG) and *slo* ^E366G^ (GGG) mRNAs from *slo* ^E366G/+^ heterozygous head tissue. (**E**) Illustration of location of SLO‐positive axonal tracts (arrowheads in **F**) in the adult nervous system. CNS, central nervous system. (**F**) SLO channel expression in the *slo* ^*loxP*/+^, *slo* ^E366G/+^, and *slo* null (*slo* ^4^) backgrounds, imaged in region noted in (**E**). Scale, 20 μm. (**G**) Illustration showing morphology of large ventral lateral neurons (l‐LN_v_s) labeled with *PDF* promoter‐driven RFP (*PDF::RFP*) and location of patch‐clamp recording sites. (**H**) Average action potential (AP) waveforms in l‐LN_v_s. Darker and lighter shades show mean and standard error of the mean (SEM). (**I–L**) l‐LN_v_ AP and afterhyperpolarization (AHP) parameters. Values of n are noted. Error bars: 95% confidence interval (CI). *P< 0.05, **P < 0.005, ***P < 0.0005, ns – *P* > 0.05, unpaired t‐test with Welch's correction (**D, I, J, L**), Mann–Whitney U test (**K**). [Color figure can be viewed at wileyonlinelibrary.com]

The D434 residue mutated in PNKD3 is located within the regulator of K^+^ conductance 1 (RCK1) domain of the channel^4^ (Figure S1A), which contains binding sites for divalent cations and connects Ca^2+^‐binding to channel opening.^13^ Consistent with its functional importance,^4^, ^5^ the D434 residue is highly conserved across Bilateria (Fig. 1A). Aspartic acid (D) appears fixed in Deuterostomes at equivalent positions to hSlo1 434, while in Protostome orthologs, including the *Drosophila* BK channel α‐subunit Slowpoke (SLO), a glutamic acid residue (E) is more prevalent (Fig. 1A). Importantly, mutating the murine equivalent of D434 (D369 in mSlo1) to E does not alter channel function over a broad range of Ca^2+^ concentrations,^5^ consistent with the similar physiochemical properties of aspartic and glutamic acid. Since the above evidence supports functional conservation of this residue between humans and *Drosophila*, we used ends‐out homologous recombination to substitute the *Drosophila* residue orthologous to hSlo1 D434 (SLO E366) with glycine (Figs. 1B,C; S1B and S2). In parallel, we isolated corresponding controls harboring the genomically encoded E residue. As part of the homologous recombination process, both lines contain a 76 base pair (bp) sequence in a non‐conserved intronic region of *slo* that includes a single *loxP* site^14^ (Fig. 1B). We isolated 10 E366G and four control alleles (Figures S1B,C, and S2), and outcrossed three of each to an isogenic iso31 strain for five generations to homogenize genetic background (see Materials and Methods). We term these alleles *slo* ^E366G^ and *slo* ^*loxP*^, respectively.

We combined RNAseq and immunofluorescent imaging to test whether the E366G mutation impacted *slo* mRNA stability or localization of the SLO channel. In *slo* ^E366G/+^ heterozygotes, RNAseq revealed no significant difference in the number of mRNA reads from the *slo* locus containing either wild‐type (GAG; +) or mutant (GGG; E366G) codons in exon 10 (Fig. 1B,D). Nor was the overall expression of *slo* between *slo* ^E366G/+^ and *slo* ^*loxP*/+^ heterozygotes (our primary experimental genotypes – see below) significantly different (log_2_ fold‐change = 0.045; *P* = 0.16; q = 0.48; n = 9 independent biological replicates/genotype). Furthermore, immunostaining of SLO channels in the adult nervous system did not reveal any obvious difference in SLO localization within axonal tracts^15^ between *slo* ^E366G/+^ and *slo* ^*loxP*/+^ flies (Fig. 1E–F). Hence, alterations in neuronal physiology and organismal behavior in *slo* ^E366G/+^ flies likely derive from changes in BK channel activity as opposed to SLO expression. Interestingly, *slo* ^E366G^ homozygotes were lethal prior to adult eclosion from the pupal case, and *slo* ^E366G/E366G^ pupae exhibited morphological abnormalities (Figure S3), suggesting a profound and dose‐dependent impact of SLO E366G channels on organismal physiology and development. We therefore focus the remainder of our studies on *slo* ^E366G/+^ heterozygotes, which are viable, fertile, and accurately model the dominant mode of inheritance of the hSlo1 D434G mutation in PNKD3.^4^

### 2.2 Altered Action Potential Shape in *slo*^E366G^ ^/+^ Neurons

As noted above, BK channels play important roles in controlling neuronal excitability by modulating AP shape and the AHP.^10^ To test how SLO E366G channels impact these neurophysiological properties, we performed ex vivo patch‐clamp recordings from adult large ventral lateral neurons (l‐LN_v_s) (Fig. 1G). These neurons are components of the *Drosophila* circadian clock network, and drive light‐dependent changes in arousal.^16^ SLO channel expression in the l‐LN_v_s oscillates in a time‐dependent manner, with high SLO channel activity observed at Zetigeber Time (ZT) 18–20 (ie, during the night) and low activity at ZT6–8 (during the day).^17^

Passive membrane properties of l‐LN_v_s were not altered in *slo* ^E366G/+^ flies at ZT18–20 (Figure S4A,B). However, analysis of spontaneous APs revealed a significant reduction in mean AP amplitude and duration, as well as enhanced AHP amplitude and accelerated AHP kinetics, in *slo* ^E366G/+^ l‐LN_v_s (Fig. 1H–L). These alterations appear to result from acute increases in SLO E366G channel expression rather than, for example, neurodevelopmental or homeostatic changes in l‐LN_v_ excitability, since we did not observe any differences in AP or AHP properties at ZT6–8, when SLO expression is low^17^ (Figure S5). Interestingly, while the D434G mutation has been hypothesized to increase neuronal firing rates by accelerating sodium channel recovery,^4^ *slo* ^E366G/+^ l‐LN_v_s did not exhibit alterations in either the rate of spontaneous firing (Figure S4C) or of higher frequency firing induced by +20 or +40 pA current injections (Figure S6) at ZT18–20, despite a narrowing of AP width (Fig. 1J).

### 2.3 SLO E366G Channels Reduce Neurotransmitter Release in a Ca^2+^‐Dependent Manner

In central presynaptic termini and neuromuscular junctions of diverse species, BK channels negatively tune neurotransmitter release by limiting activation of presynaptic voltage‐gated Ca^2+^ channels.^11^ Thus, we next investigated whether SLO E366G channels influence neurotransmitter release.

**FIG. 2:**
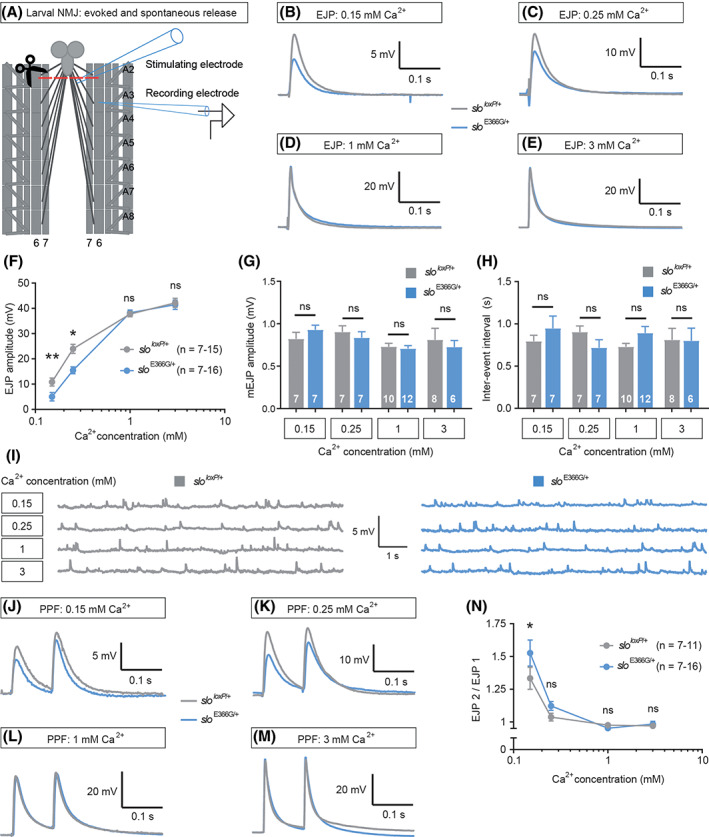
(**A**) Illustration of the electrophysiological protocol used in the larval preparation. A sharp intramuscular recording electrode records from abdominal segment A3 of the longitudinal body wall muscle 6. Motoneurons innervating the body wall muscles are severed just below the ventral nerve cord (VNC) and excitatory junction potentials (EJPs) are evoked by stimulating the severed end of the motoneurons innervating muscle 6, A3. Abdominal segments A2−A8 are shown. NMJ, neuromuscular junction. (**B–E**) Representative EJPs from *slo* ^*loxP*/+^ and *slo* ^E366G/+^ larvae at high and low [Ca^2+^]_e_. (**F**) Mean EJP amplitudes at various [Ca^2+^]_e_ in *slo* ^*loxP*/+^ and *slo* ^E366G/+^ larvae (x‐axis shown as log_10_). (**G–H**) Mean mEJP amplitude (**G**) and inter‐event interval (**H**) across a range of [Ca^2+^]_e_. (**I**) Representative mEJPs from *slo* ^*loxP*/+^ and *slo* ^E366G/+^ larvae at different [Ca^2+^]_e_. (**J–M**) Representative paired‐pulse waveforms across a range of [Ca^2+^]_e_. PPF, paired‐pulse facilitation. (**N**) Paired‐pulse ratio shown as EJP2/EJP1 at various [Ca^2+^]_e_ in *slo* ^*loxP*/+^ and *slo* ^E366G/+^ larvae (x‐axis shown as log_10_). Values of n are noted. Error bars: mean ± SEM. *P < 0.05, **P < 0.005, ns – *P* > 0.05, two‐way ANOVA with Sidak's multiple comparisons test (**F, N**). ns – *P* > 0.05, Mann–Whitney U test (**G, H**). [Color figure can be viewed at wileyonlinelibrary.com]

To do so, we turned to a highly tractable glutamatergic synapse in *Drosophila* – the 3rd instar larval neuromuscular junction^18^ (NMJ) (Fig. 2A). Neither the passive membrane properties of the muscle nor the morphology of motoneuron synapses were significantly different between *slo* ^E366G/+^ and *slo* ^*loxP*/+^ L3 larvae (Figure S7). We evoked postsynaptic excitatory junction potentials (EJPs) by severing and directly stimulating innervating motoneurons (Fig. 2A), and examined EJP amplitudes across a range of extracellular Ca^2+^ concentrations ([Ca^2+^]_e_). Since the probability of neurotransmitter release (*P*_r_) at the larval NMJ scales with AP‐evoked Ca^2+^ influx,^19^, ^20^ we used this paradigm to model the effect of SLO E366G channels on neurotransmitter release across a range of *P*_r_. At 1–3 mM [Ca^2+^]_e_ (high P_*r*_), we found no difference in EJP amplitudes between *slo* ^E366G/+^ and *slo* ^*loxP*/+^ larvae (Fig. 2B,D). In contrast, *slo* ^E366G/+^ EJPs were significantly smaller compared to *slo* ^*loxP*/+^ controls at 0.15–0.25 mM [Ca^2+^]_e_ (low P_*r*_) (Fig. 2C,D). This Ca^2+^‐dependent alteration in synaptic release was not accompanied by changes in the amplitude or frequency of spontaneously occurring miniature EJPs (mEJPs) (Fig. 2G–I), suggesting a presynaptic locus for this effect. To provide support for this premise, we examined short‐term plasticity in *slo* ^E366G/+^ larvae over the same range of [Ca^2+^]_e_. The relationship between [Ca^2+^]_e_ and short‐term plasticity is well established at this synapse. Low [Ca^2+^]_e_ results in lower vesicle release per AP, smaller EJPs, and paired‐pulse facilitation (PPF). Conversely, increasing [Ca^2+^]_e_ enhances vesicle fusion, increases EJP amplitudes, and reduces PPF.^21^, ^22^ We found that *slo* ^E366G/+^ larvae displayed a significant increase in PPF at 0.15 mM [Ca^2+^]_e_, a non‐significant trend towards an increase at 0.25 mM [Ca^2+^]_e_, and no alteration at 1 or 3 mM [Ca^2+^]_e_ (Fig. 2J–N), supporting the premise that presynaptic SLO E366G channels reduce neurotransmitter release at the NMJ at low [Ca^2+^]_e_.

### 2.4 Perturbed Pre‐Motor Circuit Function in *slo*^E366G^ ^/+^ Larvae

Collectively, the above data suggest that while SLO E366G channels do not impact neurotransmission at the NMJ under physiological conditions, they may reduce neurotransmitter release at central synapses with an intrinsically lower P_*r*._ ^23^ Thus, we next explored whether the activity of central pre‐motor circuits, which potentially harbor such synapses, were perturbed by expression of SLO E366G channels.

**FIG. 3:**
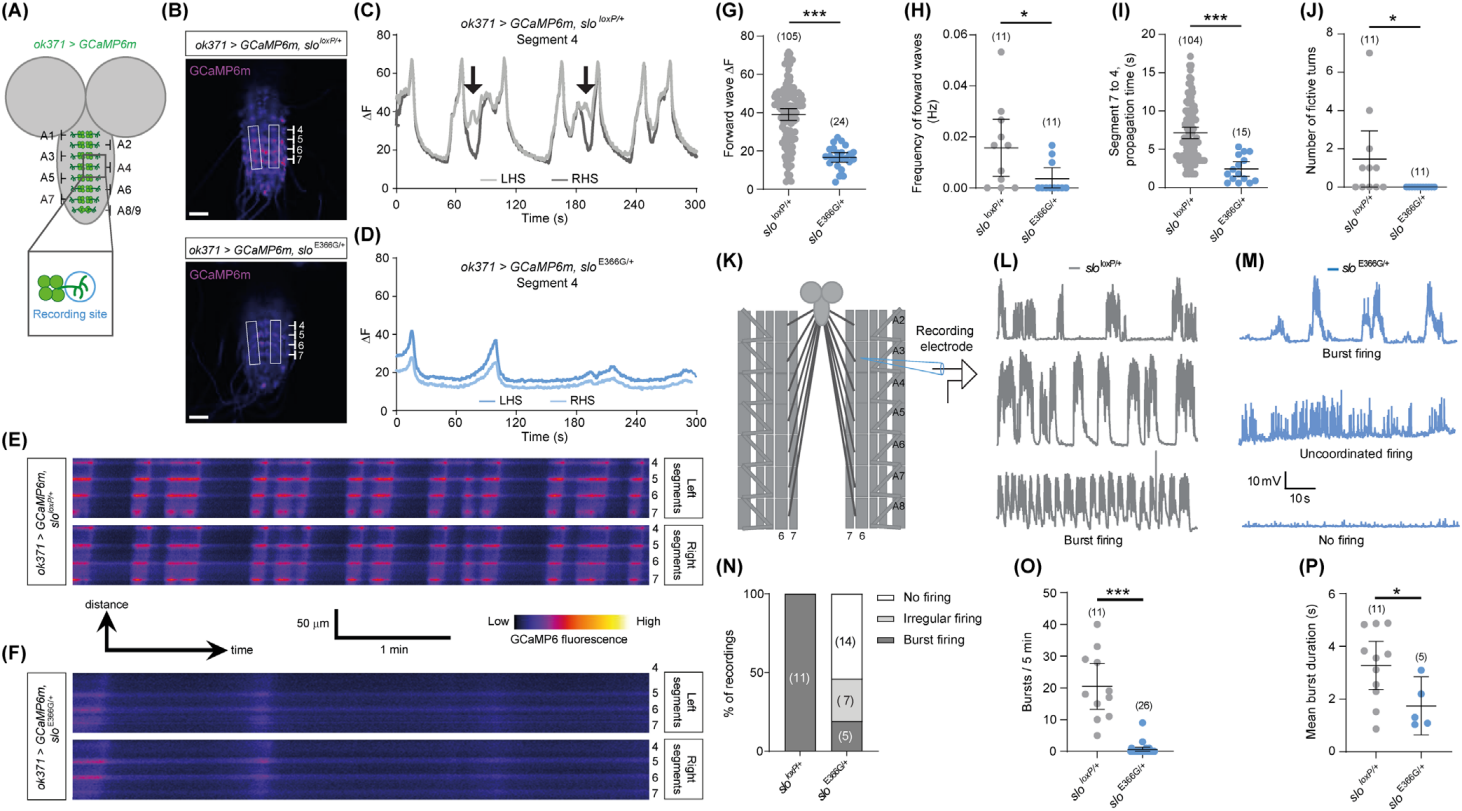
(**A**) Illustration of GCaMP6m‐labeled motoneuron cell bodies and dendritic regions in the ventral nerve cord (VNC) and location of recording area around motoneurons in the abdominal A4 segment of the VNC. (**B**) Representative images showing GCaMP6m‐labeled motoneuron cell bodies and dendrites in the VNCs of *slo* ^*loxP*/+^ and *slo* ^E366G/+^ larvae, and location of motoneuron dendrites on the left‐ (LHS) and right‐hand side (RHS) of abdominal segments 4–7. (**C, D**) Representative traces of GCaMP6m fluorescence over 300 seconds in *slo* ^*loxP*/+^ (**C**) and *slo* ^E366G/+^ (**D**) motoneuron dendrites within abdominal segment 4. Arrows in C indicate fictive turns, where the LHS and RHS motoneuron dendrites exhibit opposing patterns of excitation. (**E, F**) Line‐based kymographs showing rhythmic alterations in GCaMP6m fluorescence within dendritic domains of *slo* ^*loxP*/+^ (**E**) and *slo* ^E366G/+^ (**F**) motoneurons in abdominal segments 4–7. (**G–J**) Parameters of fictive locomotor patterns. Values of n are shown. Data are derived from n = 11 *slo* ^*loxP*/+^ and *slo* ^E366G/+^ larvae. (**K**) Illustration of the electrophysiological protocol used. Motoneuron axons innervating the body wall are left intact. Postsynaptic excitatory junction potentials (EJPs) are thus elicited via activation of motoneurons by the upstream larval central pattern generator (CPG). (**L–M**) Representative traces of spontaneous firing from *slo* ^*loxP*/+^ (**L**) and *slo* ^E366G/+^ (**M**) larvae. (**N**) Percentage of *slo* ^*loxP*/+^ and *slo* ^E366G/+^ larvae showing burst firing, irregular firing, or no firing. (**O**) Number of bursts in 5 minutes of recording. (**P**) Duration of bursts, only including recordings in which at least one burst occurred (derived from n = 11 larvae). Error bars: mean and 95% confidence interval (CI). *P < 0.05, ***P < 0.0005, Mann–Whitney U test (**G–J, O**) or unpaired t‐test with Welch's correction (**P**). [Color figure can be viewed at wileyonlinelibrary.com]

We focused on the larval central pattern generator (CPG) – an intrinsically active network that drives rhythmic excitation of motoneurons during foraging,^24^ and which is located within the abdominal and thoracic segments of the ventral nerve cord (VNC).^25^ We visualized CPG‐driven input to motoneuron dendrites in the VNCs of ex vivo larval brains by expressing a genetically encoded fluorescent Ca^2+^ sensor, GCaMP6m,^26^ under the *ok371*‐Gal4 driver, which labels motoneurons in the abdominal segments of the VNC as well as other glutamatergic neurons (which can be distinguished based on location^27^) (Fig. 3A–B). Fictive forward locomotion (CPG‐induced excitation of motoneurons that would drive locomotion) was visible as rhythmic waves of increased GCaMP6m fluorescence that moved from posterior to anterior motoneuron dendrites and cell bodies (Fig. 3C,E, and Video S1). The amplitude and frequency of posterior to anterior Ca^2+^ waves were reduced in *slo* ^E366G/+^ VNCs (Fig. 3D,F–H), while the propagation of waves from segments A7–A4 occurred more rapidly (Fig. 3I). It was also possible to identify fictive turns. Dendritic Ca^2+^ spikes were usually concurrent between motoneurons on the left and right sides of the VNC. However, as described previously,^28^ occasionally a spike in anterior segments occurred alongside a coincident trough in GCaMP6m fluorescence in the contralateral motoneuron dendrite (Fig. 3C, arrows). In behaving larvae this would result in unilateral anterior muscle contraction, facilitating a turn. Fictive turns were present in *slo* ^*loxP*/+^ controls but were not observed in *slo* ^E366G/+^ VNCs (Fig. 3C,J). These data strongly indicate that the activity of the CPG driving larval locomotion is perturbed by SLO E366G channels.

### 2.5 Aberrant Motoneuron Output and Locomotor Behavior in *slo*^E366G^ ^/+^ Larvae

To investigate the downstream consequences of CPG dysfunction in *slo* ^E366G/+^ larvae, we determined whether patterned output from motoneurons was altered by recording intramuscular voltage changes in larval muscle segments innervated by intact motoneurons (Fig. 3K). Proprioceptive neurons in the larval body wall provide sensory feedback to the CPG and/or downstream pre‐motor circuits during movement, enhancing the frequency of contractive wave propagation and thus locomotor velocity.^29^ Since we aimed to test how changes in the intrinsic activity of the CPG affected motoneuron output, independent of any potential alterations in sensory inputs, we limited proprioceptive feedback by inhibiting motoneuron‐induced muscle contractions via the voltage‐gated Ca^2+^ channel blocker nifedipine.^30^ Consistent with previous studies,^31^, ^32^ we observed periodic bursts of high‐frequency EJPs in all *slo* ^*loxP*/+^ control larvae (Fig. 3L,N). Similar bursts were observed in only a minority (5/26) of *slo* ^E366G/+^ larvae (Fig. 3M–N), and when they did occur were less frequent and of shorter duration (Fig. 3O–P). Interestingly, prior work has shown that reducing SLO expression enhances the frequency of CPG‐induced motoneuron bursts.^33^ Thus, loss and gain of BK channel function bidirectionally alter endogenous motoneuron firing in *Drosophila* larvae. Of the remaining *slo* ^E366G/+^ larvae, 14/26 showed no firing activity during 5 minutes of recording, while 7/26 showed continuous, irregular activity that was not coordinated into bursts and quiescent periods (Fig. 3N). Therefore, motoneurons in *slo* ^E366G/+^ larvae display abnormal patterns of spontaneous activity, consistent with dysfunction of the upstream movement‐driving CPG in *slo* ^E366G/+^ larvae.

**FIG. 4:**
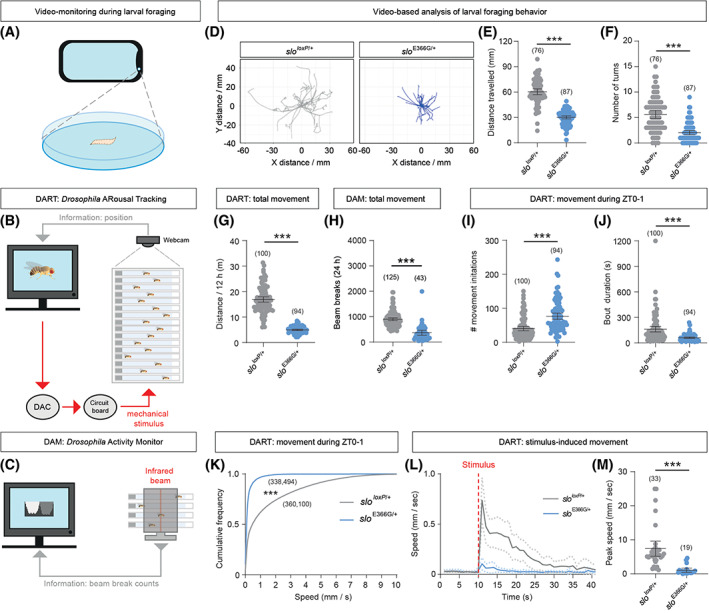
(**A‐C**) Schematics of phone‐based video‐tracking of *Drosophila* larvae foraging across an agar plate (**A**), the DART (*Drosophila* Arousal Tracking) video‐tracking system (**B**; DAC: digital to analog converter), and the DAM system (**C**), which counts breaks of an infrared beam bisecting a DAM monitor. (**D**) Overlaid traces of foraging paths traveled by individual *slo* ^*loxP*/+^ (n = 26) and *slo* ^E366G/+^ (n = 28) L3 larvae during 1 minute of free movement. (**E, F**) Mean distance traveled (**E**) and number of turns (**F**) over 1 minute between *slo* ^E366G/+^ and *slo* ^*loxP*/+^ L3 larvae. (**G, H**) Overall movement in adult male *slo* ^*loxP*/+^ and *slo* ^E366G/+^ flies measured by the DART (**G**) and DAM (*Drosophila* Activity Monitor) (**H**) systems. (**I, J**) Number (**I**) and duration (**J**) of movement bouts during 0–1 hours following lights‐on in *slo* ^*loxP*/+^ and *slo* ^E366G/+^ adult male flies. (**K**) Cumulative distribution of speeds in *slo* ^*loxP*/+^ and *slo* ^E366G/+^ males during 0–1 hours following lights‐on. Data are derived from 100 *slo* ^loxP/+^ and 94 *slo* ^E366G/+^ males. (**L**) Plot of locomotor speed in *slo* ^*loxP*/+^ and *slo* ^E366G/+^ males before and after an applied mechanical stimulus (red dotted line). (**M**) Peak speed in *slo* ^*loxP*/+^ and *slo* ^E366G/+^ males in the 1‐minute period following a mechanical stimulus. Values of n are shown. Error bars: mean and 95% confidence interval (CI). **P < 0.005, ***P < 0.0005, unpaired t‐test with Welch's correction (**E, G**), Mann–Whitney U test (**F, H–J, M**), or Kolmogorov–Smirnov test (**K**). [Color figure can be viewed at wileyonlinelibrary.com]

We next tested whether the above alterations in CPG function and patterned motoneuron output resulted in corresponding changes in larval locomotion. We measured locomotor activity in *slo* ^E366G/+^ and *slo* ^*loxP*/+^ larvae by video‐tracking 1 minute of movement across a flat agar plane (Fig. 4A).^34^ In striking agreement with the above data, we found that both the total distance moved and number of turns initiated were substantially reduced in *slo* ^E366G/+^ larvae (Figs. 4D–F and S8). These data provide proof‐of‐principle in an animal model that a BK channel GOF mutation associated with PNKD3 can alter locomotor control, and point to a central role for CPG dysfunction in generating these defects.

### 2.6 *slo*^*E366G*^ ^*/+*^ Adult Flies Exhibit Locomotor Dysfunction and Dyskinesia‐like Leg Twitches

Given that dyskinetic attacks in PNKD3 patients frequently involve involuntary movement of the extremities,^4^ we were also interested in examining how the SLO E366G mutation impacted movement in adult flies – the limbed stage of the *Drosophila* life cycle. As noted above, *slo* ^E366G/+^ adults are viable and fertile, albeit with reduced lifespan (Figure S9). While paroxysmal dystonia and chorea is co‐morbid with absence and generalized tonic–clonic seizures in some patients harboring the hSlo1 D434G mutation, we did not observe temperature‐ or bang‐induced seizures in *slo* ^E366G/+^ adults, nor ether‐induced leg shaking. However, video recordings of *slo* ^E366G/+^ adult flies revealed two clear movement‐related phenotypes. First, ~1/3 of *slo* ^E366G/+^ flies (but no *slo* ^*loxP*/+^ controls) exhibited spontaneous leg twitches across a 5‐minute period (Figure S10A,B; Video S2). Leg twitches in *slo* ^E366G/+^ males were of median duration 4 seconds but could extend to >25 seconds, and were temporally clustered into dispersed bouts (Figure S10C–L), reminiscent of paroxysmal attacks in PNKD3 patients.^4^ Second, *slo* ^E366G/+^ males appeared to exhibit an overall reduction in locomotion (Video S2). To quantify this phenotype in more detail, we used an automated video‐tracking system called DART (*Drosophila* Arousal Tracking)^35^ to monitor locomotion in adult male *slo* ^E366G/+^ and *slo* ^*loxP*/+^ flies over a 12‐hour period (Fig. 4B). *slo* ^E366G/+^adult males indeed exhibited a robust reduction in distance traveled compared to controls (Figs. 4G and S11). Using a distinct, more high‐throughput, activity monitoring system (the *Drosophila* Activity Monitor; DAM^36^) (Fig. 4C), we confirmed reduced locomotion in *slo* ^E366G/+^ adult males (Fig. 4H) and found that such movement defects are apparent across the day/night cycle (Figure S12), showing that reduced movement in *slo* ^E366G/+^ males is not an artefact caused by perturbed circadian rhythms.^37^

Since male flies are inactive for much of the day,^38^ we performed a more detailed analysis of movement during a period of normally heightened activity: 0–1 hours following lights‐on in 12 hours light: 12 hours dark conditions. During this time span, DART recordings revealed that *slo* ^E366G/+^ males initiate movement more frequently than controls (Fig. 4I). However, the duration of locomotor bouts was shorter (Fig. 4J), and overall locomotor speeds significantly reduced (Fig. 4K), in *slo* ^E366G/+^ males. Similar results were observed in *slo* ^E366G/+^ adult females (Figure S13). We also used the DART system to apply a mechanical stimulus to quiescent *slo* ^E366G/+^ and *slo* ^*loxP*/+^ males (see Materials and Methods), and observed that the speed of stimulus‐induced movement was strongly reduced in *slo* ^E366G/+^ males (Fig. 4L–M). Thus, the SLO E366G mutation impairs self‐driven movement in larval and adult *Drosophila*, and stimulus‐induced movement in adult *Drosophila*.

### 2.7 Cell‐Specific Induction of SLO E366G Channels Suggests a Pathogenic Role in Cholinergic Neurons

**FIG. 5:**
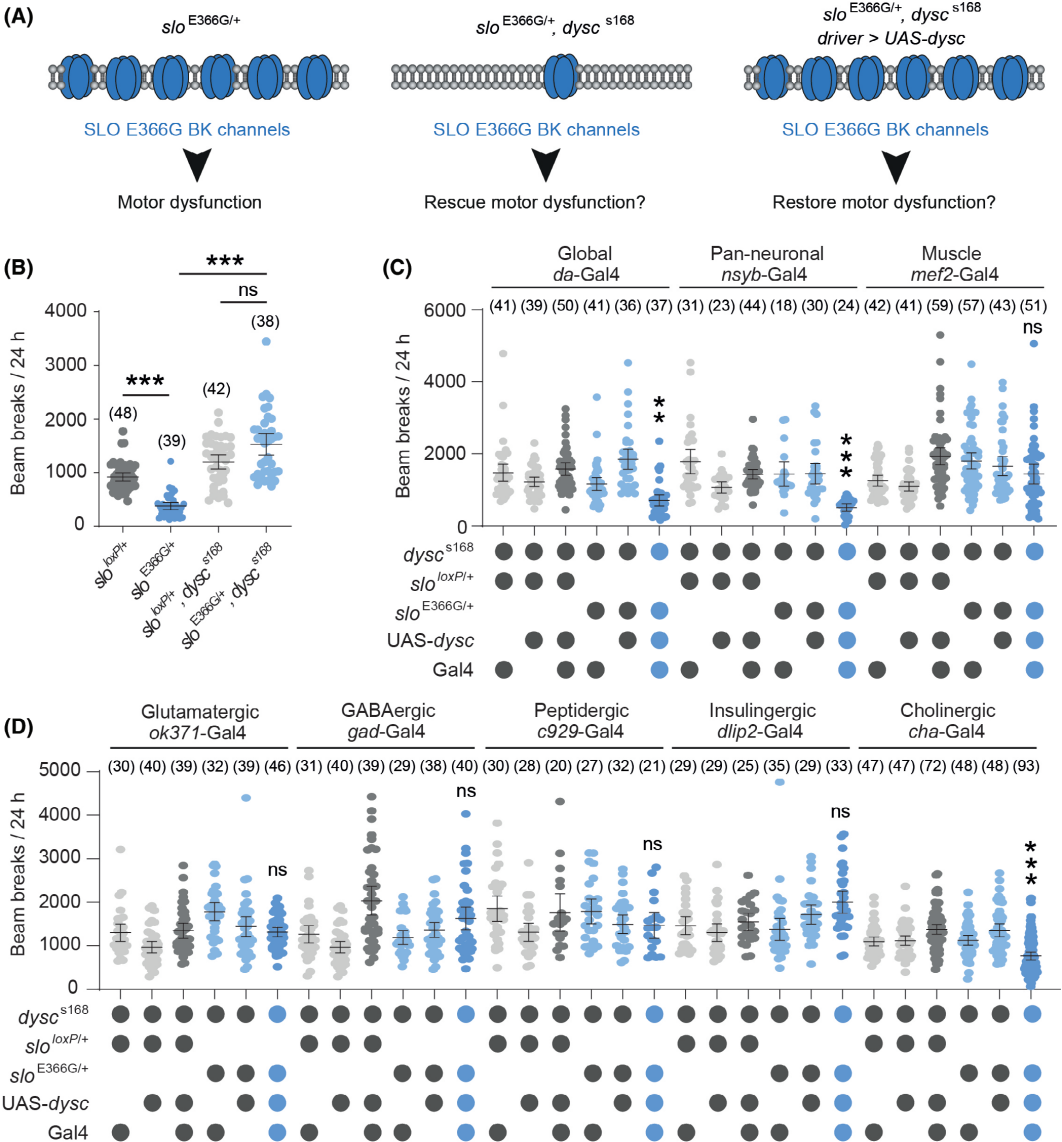
(**A**) Model of the *dysc*‐based system for indirect, cell‐specific control of SLO E366G expression. (**B**) Locomotor activity in *slo* ^E366G/+^ and *slo* ^*loxP*/+^ adult males with wild‐type or loss‐of‐function (*dysc* ^s168^) *dysc* alleles. (**C, D**) Cell type‐specific restoration of DYSC in *slo*, *dysc* double mutants. Cell types associated with each *promoter*‐Gal4 driver are shown. Columns of blue circles denote the experimental genotypes in each dataset. Smaller dots in each graph represent measurements derived from individual adults. Values of n are noted. Error bars: mean and 95% confidence interval (CI). **P < 0.005, ***P < 0.0005, Kruskal−Wallis test with Dunn's post hoc test. [Color figure can be viewed at wileyonlinelibrary.com]

BK channels are broadly expressed in both neuronal and non‐neuronal tissues,^8^ and the cell‐types in which GOF BK channels act to impact movement are unknown. To begin to identify such cell types, we developed a genetic system that allowed us to limit robust SLO expression to specific cell types of interest. This method is based upon a gene called *dyschronic* (*dysc*), orthologous to the human deaf‐blindness gene *Whirlin/DFNB31*.^15^, ^39^ *dysc* encodes a scaffold protein (DYSC) that promotes neuronal SLO channel expression. *dysc* loss‐of‐function (LOF) mutants exhibit greatly reduced neuronal SLO expression and arrhythmic patterns of locomotion across the day–night cycle, but no significant disruption in overall motor capacity.^15^ If the SLO E366G mutation indeed causes GOF, we predicted that coincident homozygosity for the LOF *dysc* ^s168^ allele would suppress movement defects in *slo* ^E336G/+^ adults, since GOF BK channels would not be robustly expressed (Fig. 5A). Using the DAM system, we found that this was the case (Fig. 5B). We were then able to restore DYSC (and thus SLO E366G) expression in *slo* ^E366G/+^, *dysc* ^s168^ double mutants by combining a UAS‐*dysc* transgene with the UAS‐binding Gal4 transcription factor under control of a cell‐specific promoter. This approach yields robust SLO E366G expression in defined cell types, allowing us to test whether this causes movement defects. We found that global or pan‐neuronal (but not muscle‐specific) SLO E366G expression strongly reduced movement (Fig. 5C) and, furthermore, that inducing SLO E366G expression solely in cholinergic (but not glutamatergic, GABAergic, peptidergic, or insulinergic) neurons also reduced movement, albeit with lower penetrance relative to pan‐neuronal restoration (Fig. 5D). Thus, SLO E366G channels act in neurons to perturb adult movement, and this effect partially maps to cholinergic neurons.

## 3 Discussion

PNKD3 has been linked to GOF mutations in the hSlo1 BK channel.^3^, ^4^, ^5^, ^7^, ^40^ Yet, due to a lack of animal models of PNKD3, how pathologically enhanced BK channel activity impacts neuronal excitability, synaptic plasticity, and pre‐motor circuit activity in vivo has been unclear. In addition, whether equivalent mutations to hSlo1 D434G are capable of modifying movement in non‐human species has remained unexplored. Here we address these issues using a *Drosophila* knock‐in model of PNKD3.

Although highly homologous, *Drosophila* BK channels exhibit a lower Ca^2+^‐ and voltage‐sensitivity compared to their mammalian counterparts when expressed in *Xenopus* oocytes.^41^ Furthermore, we have not directly measured BK channel currents in SLO‐expressing neurons of *slo* ^E366G/+^ flies. Thus, the precise biophysical impact of the SLO E366G mutation on BK channel function remains to be fully determined. Nonetheless, several lines of evidence presented herein indicate that SLO E366G causes GOF, similarly to hSlo1 D434G, and enhances the net activity of BK channels in vivo. First, LOF mutations in mammalian and *Drosophila* BK channel α‐subunits result in a broadening of APs and a loss of the AHP current in BK channel‐expressing neurons.^33^, ^42^ However, in *slo* ^E366G/+^ l‐LNv neurons, the reverse is true: AP width is narrowed and AHP amplitude is enhanced. Second, BK channel LOF enhances neurotransmitter release at the frog, *Caenorhabditis elegans*, and *Drosophila*, NMJs.^39^, ^43^, ^44^ In contrast, we observed a reduction in neurotransmitter release solely at low [Ca^2+^]_e_ at the *slo* ^E366G/+^ larval NMJ. Third, the pronounced reduction in movement in *slo* ^E366G/+^ adults was suppressed by loss of DYSC, a scaffold protein that binds SLO and promotes SLO expression in fly neurons.^15^ Thus, while we cannot entirely rule out a neomorphic effect of the SLO E366G mutation, our data are consistent with SLO E366G acting as a dominant, GOF mutation. The specific impact of SLO E366G channels on neurotransmission at low [Ca^2+^]_e_ leads us to speculate that the GOF effect of SLO E366G likely involves an increase in BK channel Ca^2+^‐sensitivity, similarly to the effects of an equivalent mutation to hSlo1 D434G in the murine BK channel α‐subunit.^5^ Furthermore, the multifaceted defects in motor control combined with pre‐motor circuit dysfunction in *slo* ^E366G/+^ larvae and adult flies support the genetic linkage between the hSlo1 D434G mutation and dyskinesia,^4^ and demonstrate that BK channels play conserved roles in regulating movement across distantly related bilateral species.

Collectively, our analyses of neurotransmission at the larval NMJ, activity of the larval CPG, and the partial mapping of locomotor dysfunction in *slo* ^E366G/+^ adult flies to cholinergic neurons suggests a model in which SLO E366G channels impair movement by at least two potentially overlapping mechanisms: first, by perturbing the intrinsic activity of CPGs that provide patterned excitatory input to motoneurons; and second, by reducing acetylcholine release from cholinergic pre‐motor circuits. Thus, while altered activity of cerebellar and basal ganglia‐thalamocortical circuits are common hallmarks of PxDs,^45^, ^46^ our findings suggest two particular pre‐motor circuits that will be interesting to examine in future vertebrate models of PNKD3. First, spinal CPG networks that transform descending command signals from brain regions such as the mesencephalic locomotor region into coordinated movements,^47^ and second, striatal cholinergic interneurons (ChIs). This latter cell type is a particularly intriguing candidate for contributing to involuntary movements in PNKD3. BK channels in ChIs regulate AP repolarization,^42^ and thus potentially impact neurotransmitter release from this cell type. Furthermore, acetylcholine release from ChIs modulates numerous striatal and neostriatal cell types via muscarinic and nicotinic acetylcholine receptors, including glutamatergic cortical afferents, GABAergic medium spiny output neurons, and GABAergic/dopaminergic interneurons; and aberrant patterns of striatal output are thought to contribute to involuntary movements in dystonia and dyskinesia.^48^

However, it is important to note that our results suggest that SLO E366G channels act in more than one neural cell type to impair movement. Thus, it will be interesting to extend our cell‐specific mapping approach to examine the effect of expressing SLO E366G channels in other neuromodulatory circuits (dopaminergic, serotonergic, etc.), as well as specific pre‐motor neuropil domains in the *Drosophila* brain. It is also notable that, in contrast to *slo* ^E366G/+^ flies, gross locomotor ability in PNKD3 patients is largely normal, with dyskinetic attacks generally triggered by alcohol, stress, or fatigue.^4^ Such differences in phenotypic penetrance and severity due to orthologous *Drosophila* and human mutations may arise through a number of mechanisms, including the absence of BK channel β‐subunits in *Drosophila* (which modulate the biophysical impact of the D434G mutation^3^, ^12^), differences in the repertoire of ion channels contributing to AP shape and neurotransmitter release, divergent use of acetylcholine (*Drosophila*) and glutamate (vertebrates) as predominant excitatory neurotransmitters, and species‐specific variations in neural circuit architecture. Nonetheless, the robust motor defects observed in *slo* ^E366G/+^ flies are an advantageous aspect of this model, since automated activity‐monitoring systems can now be deployed in concert with classical genetics to search for conserved genetic modifiers of motor dysfunction in this model. In contrast, involuntary movements in mouse models of inherited dyskinesia/dystonia frequently require stress or drug injections to induce their occurrence,^49^, ^50^ limiting their utility for high‐throughput phenotype‐based screens.

Our *Drosophila* model thus provides a rapid platform to define key neuronal subtypes and cellular pathways via which GOF BK channels alter motor control, and identify genetic perturbations capable of modifying phenotypic severity. It will also be interesting to utilize *Drosophila* models to study other *KCNMA1*/hSlo1 mutations linked to PNKD3,^6^, ^7^ as well as LOF mutations in *KCNMA1* linked to ataxia.^51^, ^52^

## Author Roles

Conceptualization: P.K., S.L., J.E.C.J. Methodology: P.K., E.B., K.‐F.C., A.P., S.L., J.E.C.J. Software: P.K. Validation: P.K., S.L., E.B. Formal Analysis: P.K., S.L., E.B., J.E.C.J. Investigation: P.K., S.L., E.B., K.‐F.C., S.L., J.E.C.J. Writing – Original Draft: J.E.C.J. Writing – Review and Editing: P.K., E.B., K.‐F.C., S.L., D.M.K., J.J.L.H., J.E.C.J. Visualization: P.K., S.L., E.B., J.E.C.J. Supervision: D.M.K., J.J.L.H., J.E.C.J. Project Administration: J.J.L.H., J.E.C.J. Funding Acquisition: P.K., D.M.K., J.J.L.H., J.E.C.J.

## Supporting information


**Appendix S1:** Supplementary Information

**Video S1** Video shows representative examples of waves of motoneuron excitation in the ex vivo larval ventral nerve cord (VNC) visualized via GCaMP6m driven by the glutamatergic neuron driver *ok371*‐Ga4. In the *slo* ^*loxP*/+^ control VNC (left), repetitive waves of excitation traveling from posterior to anterior segments can clearly be observed. Unilateral motoneuron excitation in the upper segments can also be infrequently observed. In the *slo* ^E366G/+^ VNC (right), both the magnitude and frequency of increases in motoneuron GCaMP6m fluorescence is reduced, indicative of perturbed input from the upstream central pattern generator.

**Video S2** Video shows representative examples of three *slo* ^*loxP*/+^ (left) or *slo* ^E366G/+^ (right) adult male flies freely moving in glass tubes containing an agar‐sucrose food source. Tubes are horizontally placed. Note the more rapid speed and increased continuity of movement of *slo* ^*loxP*/+^ compared to *slo* ^E366G/+^ males. Subsequent zoom of the left‐hand *slo* ^E366G/+^ male illustrates a bout of unilateral leg twitches, similar instances of which were frequently observed in *slo* ^E366G/+^ but not *slo* ^*loxP*/+^ flies.
