# Supplementary material for: Impaired Pre‐Motor Circuit Activity and Movement in a Drosophila Model of KCNMA1‐Linked Dyskinesia
Source: Mov Disord. 2021 Jan 15;36(5):1158–69. doi: 10.1002/mds.28479 (PMC8248399; doi:10.1002/mds.28479)
Supplement: Supplementary file 1 — Appendix S1: Supplementary Information [file MDS-36-1158-s001.docx]

**Impaired pre-motor circuit activity and movement in a *Drosophila* model of *KCNMA1*-linked dyskinesia**

Patrick Kratschmer PhD, Simon A. Lowe PhD, Edgar Buhl PhD, Ko-Fan Chen PhD, Dimitri M. Kullmann DPhil FMedSci, Alan Pittman PhD, James J.L. Hodge PhD, and James E.C. Jepson DPhil

**A. Supplemental Materials and Methods**

**B. Supplemental Figures, Figure legends, and Video Legends**

**C. Supplemental References**

**A. Supplemental Materials and Methods**

***Drosophila* husbandry**

Flies were maintained on standard fly food at a constant temperature (25°C) under 12 h: 12 h light-dark cycles (12L: 12D). The following strains were obtained from the Bloomington *Drosophila* stock center (BDSC): *y, w; hs-FLP, hs-I-SceI*/CyO (BDSC #6934)*, y, w, ey-FLP* (BDSC #5580) and *y, w, Cre; +; D*/*TM3, *sb* (BDSC #851)*.* The isogenic iso31 wild type strain used for outcrossing and *dysc*^s168^ mutants were kind gifts from Prof. Kyunghee Koh (Thomas Jefferson University). *PDF::RFP* was a kind gift from Prof. Justin Blau (New York University). A recombinant of the *ok371*-Gal4 driver and the UAS-*GCaMP6m* Ca^2+^ indicator was a kind gift from Dr. Stefan Pulver (University of St. Andrews).

**Generation of the *slo*^E366G^ and *slo^loxP^* alleles**

Ends-out homologous recombination was performed as described previously^1^. Recombinogenic arms corresponding to the *slo* locus were amplified using the following primers: Arm 1 forward – CGTACGTCCCCAAGTACAGACAGCAA, Arm 1 reverse – GGCGCGCCGTTGTCAGTGTGTCGTGTGC; Arm 2 forward – GGTACCGCAGCTCAATGGAATGTGATT, Arm 2 reverse – GCGGCCGCACGCTTATTCTGGGACTTCG. Underlined sequences indicate restriction enzyme cut sites. The above primers amplify 2491 bp 5’ of position chr3R:24,686,818 (*Drosophila melanogaster* Genome Assembly BDGP6) for Arm 1 and 2731 bp 3’ of position chr3R:24,686,862 for Arm 2. The A1097G point mutation resulting in the E366G amino-acid change was introduced into Arm 2 via a customised DNA fragment (GeneArt Gene Synthesis, ThermoFisher Scientific) that was used to replace the 5’ 722 bp of Arm 2. Arms were cloned into *p[w25.2]* using standard techniques. Embryonic injection of the *p[w25.2]*-Arm2^E366G^-Arm1 vector was performed by BestGene Inc. (CA, USA). Primers used to validate successful recombination events are as follows: pW-Acc3_F (forward primer) – GCTCAGCTTGCTTCGCGATGTGTTCAC, pW-Acc1_R (reverse primer) – TTAGTTGAGTGCTTAAATTCAAAGGAT. The presence or absence of the A1097G point mutation in Arm 2 was verified via Sanger sequencing using the following primers: a2_alternative_R2-validation_F (R2F) – TCCGCTTTAATCGCACACTA; GEPD_seq_1 – CCCCCACCTTCAACAACACA. We also sequenced the entirety of Arm 1 and Arm 2 post-recombination and did not find any additional sequence variants in these regions apart from previously described intronic polymorphisms^2 3^. To outcross each allele into an isogenic background, the following primers were used: OCF (forward primer) *–* AGACTAGTCTAGGGTACCGCA, OCR (reverse primer) – TAGTTCCTTGAATTGGCAGCG. OCF binds to a portion of the 76 bp of sequence incorporated into the intron upstream of exon 10 (which includes the single *loxP* site), and therefore will only generate a product in a *slo*^E366G/+^ or *slo^loxP^*^/+^ background. Behavioural analyses of larval and adult movement shown in Figs. 4, and Supplemental Fig. 13B-E were obtained from heterozygotes pooled from three independently derived and outcrossed *slo*^E366G^ or *slo^loxP^* alleles. Data from corresponding individual alleles are shown in Supplemental Figs. 8, 11, and 13A. All remaining data was derived from single outcrossed *slo*^E366G^ and *slo^loxP^* recombinant alleles (25.1.1 and 132.1.1, respectively; Supplemental Fig. 1 and 2)

**Bioinformatics**

hSlo1 orthologs were initially identified using BLASTp (https://blast.ncbi.nlm.nih.gov/Blast.cgi?PAGE=Proteins), using either the hSlo1 21 amino-acid sequence shown in Fig. 1A or full-length *Drosophila* SLO as queries. Orthologous hits defined as encoding Ca^2+^-activated K^+^ channels were aligned in Clustal Omega (https://www.ebi.ac.uk/Tools/msa/clustalo/). Alignments were visualised using BoxShade (https://embnet.vital-it.ch/software/BOX_form.html), with 70% of sequences required to agree for shading. Black shading indicates amino-acid sequence conservation; grey shading indicates functional conservation.

**RNAseq**

Total RNA from3-6 day old *slo*^E366G/+^ and *slo^loxP^*^/^*^+^* adult male heads was extracted using standard techniques (9 independent biological replicates for each genotype). RNA integrity was measured via on-chip gel electrophoresis (Agilent 2100 Bioanalyzer: Agilent Technologies). cDNA libraries were prepared from mRNA using the KAPA mRNA HyperPrep Kit (KAPA Biosystems Inc., KK8580), and KAPA Single-Indexed Adapter Sets A and B (KAPA Biosystems Inc., KK8710) were used to ligate adapter sequences. cDNA libraries were sequenced via paired-end sequencing on an Illumina HiSeq 3000 sequencing system. Resulting SAM files were converted to BAM files (including BAI files) using SAMtools (v. 1.9), and the Integrative Genomics Viewer (IGV) was used to visualise RNA-seq alignments, using BAM and BAI files as inputs. The DESeq2 R package was used identify differentially expressed genes (DEGs)^4^. In this work, we use this dataset to determine whether the E366G mutation alters *slo* mRNA expression; DEGs identified through the above pipeline will be described in a subsequent manuscript.

**Immuno-histochemistry**

Adult male brains (3-7 days old) were dissected and immuno-stained as described previously^5^. Brains were fixed using Bouins solution (Sigma Aldrich) and blocked in 5% normal goat serum. A rabbit polyclonal antibody against hSlo1 was used to immuno-label *Drosophila* SLO (Abcam, UK; ab3586). This antibody was raised against a peptide sequence from hSlo1 (TELVNDTNVQFLDQDDD; amino-acids 945-961) that is 94% identical in *Drosophila* SLO (TELVNDSNVQFLDQDDD; single amino-acid mis-match is underlined). The absence of axonal immuno-staining in *slo* null flies (*slo*^4^ homozygotes; Fig 1F), observed previously using a distinct anti-SLO antibody^6^, confirms the specificity of the ab3586 antibody (note that dispersed, punctate, non-specific signals in neuropil regions throughout the adult brain were also observed in all genotypes). Brains were incubated in primary antibody (ab3586, 1:200) overnight at 4°C. Fluorescently conjugated secondary antibody (goat anti-rabbit Cy5; Invitrogen) was used at 1:1000, again via overnight incubation at 4°C. Brains were mounted and images in SlowFade Gold anti-fade mountant (Thermofisher).

**Patch-clamp electrophysiology**

Whole-cell patch clamp recordings were performed on large lateral ventral neurons (l-LN_v_) as described previously^7^. To visualize the neurons a *PDF::RFP*  fusion construct^8^ was used and crossed to *slo^loxP/loxP^* homozygotes for control and *slo*^E366G^*/TM6b*, *tb* for the experimental genotype. Two- to five-day old male flies were decapitated at Zeitgeber Time (ZT) 18-20 (i.e. 6-8 h after lights-off) under red light illumination, or at ZT6-8 under normal light illumination, and brains dissected in external solution (in mM: 101 NaCl, 1 CaCl_2_, 4 MgCl_2_, 3 KCl, 5 glucose, 1.25 NaH_2_PO_4_, 20.7 NaHCO_3_, pH 7.2). This time-point has previously been shown to represent a period of maximal SLO expression in the l-LN_v_s^9^. After cleaning and removal of the ganglion sheath, brains were placed in the recording chamber ventral side up and secured using a custom-made harp. Recordings were made at room temperature (20-22°C) with borosilicate glass electrodes (8-15 MΩ resistance) filled with internal solution (in mM: 102 K-gluconate, 17 NaCl, 0.94 EGTA, 8.5 HEPES, 0.085 CaCl_2_, 1.7 MgCl_2_, pH 7.2). Signals were amplified (Axon MultiClamp 700B), digitized (Axon DigiData 1440A; sampling rate: 20 kHz; filter: Bessel 10 kHz), recorded (pClamp 10, Molecular Devices, Sunnyvale, CA, USA) and the liquid junction potential (13 mV) subtracted from the membrane voltages before analysis. The resting membrane potential (RMP) and spontaneous firing rate (SFR) were measured after allowing the recordings to stabilize for one minute. Input resistance was calculated using Ohm’s law by measuring the voltage change in response to hyperpolarizing currents and excitability was measured by injecting depolarizing current pulses (0-40 pA, 5 pA increments) of either 1 s or 5 s duration. Cells were held at the RMP during current injection. The action potential size (AP peak), width at half maximal amplitude (half-width), afterhyperpolarization (AHP) amplitude and the time from peak to AHP were measured from averages of 10 spikes for each recording, aligned to the peak and measured relative to RMP.

**NMJ electrophysiology**

*Standard recordings*

Sharp-electrode intramuscular voltage recordings were taken from muscle 6, abdominal segment 3 according to standard protocols^10^. Wandering L3 larvae were dissected in ice-cold, Ca^2+^-free modified HL3.1 saline^11^ consisting of 70 mM NaCl, 5 mM KCl, 10 mM NaHCO_3_, 115 mM sucrose, 5 mM trehalose, 5 mM HEPES, and 10 mM MgCl_2_. Motor nerves were severed just below the VNC, and the brain was removed. CaCl_2_ was added to the bath solution at the concentrations indicated. Recordings took place at 22-25°C. Sharp microelectrodes (thick-walled borosilicate glass capillaries, pulled on a Sutter Flaming/Brown P-97 micropipette puller) were filled with 3 M KCl and had resistances of 20–30 MΩ. Recordings were amplified using an Axon Instruments AxoClamp-2B, digitised at 25 kHz using a National Instruments DAQ, and analysed offline using StrathClyde Electrophysiology Software WinEDR v3.2.7. Input resistance was calculated by injecting a step current of 1 nA for 500 ms and deriving the resistance from the voltage change using Ohm’s law, with the pipette resistance subtracted by bridge balancing. Recordings were discarded if their initial RMP was more positive than -60mV, varied by > 10% during recording, or if the input resistance was < 5 MΩ. EJPs were evoked by drawing the severed ends of motoneurons into a thin-walled glass suction electrode and stimulating with a single square-wave voltage pulse of 0.1 ms and 10 V (Digitimer DS3 Isolated Current Stimulator). To analyse EJP amplitude, motoneurons were stimulated 20 times at 5 s intervals, and the mean amplitude was taken. We present EJPs values that are not corrected for non-linear summation, which may potentially enhance the similarity of EJPs between *slo*^loxP/+^ and *slo*^E366G/+^ larvae at high [Ca^2+^]_e_^12^. To analyse paired pulses, motoneurons were stimulated twice with a 0.1 s interval, and this was repeated 5 times at 10 s intervals. The amplitude of the second event was calculated as a percentage of the first, and the mean percentage was taken. Spontaneously occurring mEJPs were automatically identified using WinEDR during 150 s recording in the absence of stimulation, and verified manually. Mean mEJP amplitudes and inter-event intervals were taken from at least 100 events per recording.

*Semi-intact recordings*

The procedure was as above except the brain and motor nerves were left intact and care was taken not to damage them during dissection. Recordings were performed in HL3.1 saline consisting of 70 mM NaCl, 5 mM KCl, 10 mM NaHCO_3_, 115 mM sucrose, 5 mM trehalose, 5 mM HEPES, 1 mM Ca^2+^, and 10 mM MgCl_2_. To limit muscle contractions, 25 µM nifedipine (Sigma Aldrich) was added to the bath solution^13^. Nifedipine was pre-dissolved in DMSO, which was added to the solution with a final concentration of 0.2%. Each recording lasted 300 s in the absence of stimulation. Due to the high level of activity it was not possible to measure input resistance, so to ensure high fidelity recordings were discarded if the RMP did not remain more negative than -60 mV for the duration of recording. To analyse burst firing, a ‘burst’ was arbitrarily defined as starting when at least five EJPs occurred in succession at > 5 Hz (i.e. 5 successive inter-EJP intervals of < 0.2 s), and ending when at least 1 s passed with no EJPs occurring at > 5 Hz (i.e. no inter-EJP intervals of < 0.2 s).

**NMJ morphology**

Immunostaining of larval NMJ was performed according to standard protocols^5^. Wandering L3 larva were dissected as above and fixed in 4% PFA for 10-20 mins at room temperature. Primary antibodies were goat anti-HRP conjugated Alexa Fluor 488 at 1:500 (Jackson ImmunoResearch) and mouse anti-DLG at 1:200 (clone 4F3, DSHB). Secondary antibody was goat anti-mouse Alexa Fluor 555 at 1:1000. NMJs innervating muscle 6/7, abdominal segment 3 were visualised using a Zeiss confocal LSM710 with an EC ‘Plan-Neofluar’ 20x/0.50 M27 air objective. Bouton number was counted manually and included both 1b and 1s type boutons. Bouton size, NMJ area and muscle area were quantified using ImageJ. All analyses were performed blind to genotype.

**GCaMP6m imaging**

Age-matched L3 larvae of genotypes *ok371*-Gal4 > UAS-*GCaMP6m*, slo^E366G/+^ and *ok371*-Gal4 > UAS-*GCaMP6m*, slo*^loxP^*^/+^ were obtained by crossing *ok371*-Gal4, UAS-*GCaMP6m* recombinants to *slo^loxP^*/TM6b, *tb* or *slo*^E366G^/TM6b, *tb* flies, with non-*tb* 3^rd^ instar larvae subsequently selected for experiments. Larvae were dissected in a recording solution consisting of 135 mM NaCl, 5 mM KCl, 4 mM MgCl2, 2 mM CaCl2, 5 mM TES, and 36 mM sucrose^14^. The entire CNS was removed and immersed in the recording solution. Recordings were taken immediately after dissection on a Zeiss LSM 710 confocal microscope with an EC ‘Plan-Neofluar’ 20x/0.50 M27 air objective. Recordings lasted 5 min with a scan time of 390.98 ms per frame and 200 ms intervals between scans. Fluorescence activity was measured within regions of interest (ROIs) of consistent area (115.809 µm^2^) drawn around the dendritic regions of motoneurons in segments 7 and 4 on each side of the VNC. Background fluorescence was subtracted from each of these 4 datasets using an extra ROI drawn in the top left corner of the image lacking any neural tissue. Data from the four ROIs were plotted separately. Spikes in fluorescence were identified manually. ‘Forward waves’ were defined as concurrent spikes on both sides of segment 4 which were preceded by concurrent spikes on both sides of segment 7. ‘Forward wave ΔF’ was defined as the distance from peak to trough fluorescence of a segment 4 forward Ca^2+^ wave. ‘Frequency of forward wave’ was defined as the number of segment 4 forward wave peaks per second during each 5 min recording period. ‘Propagation time’ was defined as time between segment 7 and 4 peaks during a forward wave. ‘Turns’ were defined as asymmetric spikes occurring on only one side of segment 4. Kymographs were generated using the Kymograph builder plugin in FIJI. A 100 µm line spanning regions containing motoneuron dendrites within abdominal segments 4-7 (on each side of the larval ventral nerve cord) was used as the region of interest. Total duration of each kymograph is 5 min.

**Larval locomotor analysis**

Age-matched ‘wandering’ third instar (L3) larvae were selected, gently cleaned with Milli-Q water, and transferred individually to a wide flat plane coated in 2% agar (Sigma-Aldrich), which was placed in an incubator (LSM) at 25ºC and a relative humidity of 50-55%. For each larva, after 10 s of acclimatization, 60 s of free movement was recorded using a Samsung S5 mobile phone at 15 fps and 640x480 resolution. Video-tracking was performed using the desktop version of the AnimApp video tracker^15^, available on https://github.com/sraorao/. A customized R script (R v. 3.6.0), which has been made available on GitHub (https://github.com/PatrickKratsch/AnimApp_analysis), was used to calculate the following movement parameters: total distance travelled was calculated by summing the difference in XY position between consecutive frames; turns were identified by calculating changes in larval width:length ratio, with a threshold ratio of 0.4 manually identified as a reliable indicator of turning. There was no significant difference between the three control or experimental lines in either parameter: thus, the data were pooled. In subsequent electrophysiological and Ca^2+^ imaging experiments, one control (132.1.1) and one experimental (25.1.1) line were used.

**Adult behavioural analyses**

For adult-stage behavioural analyses, three- to seven-day old male or female flies were collected and loaded into glass tubes (Trikinetics inc., MA, USA) containing 4% sucrose and 2% agar (w/v). Locomotor activity was recorded either using the Drosophila ARousal Tracking (DART, BFKlab, UK) system^16^, or the *Drosophila* Activity Monitor (DAM, Trikinetics inc., MA, USA) in 12L: 12D at 25^°^C as described previously^17^. All DART datasets were derived from ≥ 3 independent biological replicates. A customised R script was used to analyse movement parameters derived from the DART video-tracking data, available on GitHub: https://github.com/PatrickKratsch/DART_analysR. This script first creates an ‘offset table’ from the DART tracking data, obtained by subtracting successive x- and y-values. Hence, the offset table defines the displacement for each fly between consecutive frames. The total distance travelled between consecutive frames was calculated for each fly. Summing all frame-to-frame displacements over 12 h yielded the total distance travelled during this period. Flies that did not move during the second half of the experiment (hours 7 to 12) were identified as dead and excluded from further analysis. Of note, the precise duration analysed was 716 min (11 h and 56 min) due to the removal of 2 min post-lights-on and 2 min pre-lights-off, during which the lighting conditions prevented appropriate video-tracking.

Analyses of other movement parameters are based on a period of 1 h after the dark-light transition (9:02-10:02 am), a time of robust activity in wild-type flies. To analyse the distribution of locomotor speed, empirical cumulative distribution plots were generated based on 1 s movement bins (3601 bins for a 1 h and 1 s period). To analyse movement initiation, a sliding window of length 2 quantified how often a 1 s bin of non-movement was followed by a 1 s bin of movement. To analyse mean movement bout length, the fly movement data were transformed into a binary matrix, with 1’s indicating movement and 0’s non-movement. Subsequently, the lengths of movement streaks (streaks of 1’s) were extracted and averaged.

For experiments involving mechanical stimulation of *slo^loxP^*^/+^ and *slo*^E366G/+^ adult flies, a Single vibrational stimulus was delivered at ZT3, consisting of a train of 5 200ms vibrational pulses with 800ms inter-pulse intervals, set at the DART system's maximum intensity. Speed (mm/s) was analysed by the DART system from absolute position, and binned in one minute intervals. Flies were only included in analysis if they were quiescent at the time of the stimulus, defined as not exceeding an average of 1 mm/s speed in the five one-minute bins preceding the stimulus. Responses were quantified as the speed of each fly in the one minute bin immediately following the stimulus.

To quantify spontaneous leg-twitches, manually recorded videos were taken using an iPad Pro (Apple, CA, USA). Glass tubes (Trikinetics) were placed horizontally to avoid any potential confounding effects of possible alterations in geotaxis. To ensure that our analysis did not conflate ‘dyskinetic movements’ with other motor behaviors such as grooming or locomotion, leg twitches were scored using the following criteria: 1. That leg ‘twitches’ occurred only in a single limb; instances where >1 limb exhibited simultaneous movement were not scored. 2. That each bout consisted of >1 repetitive movements of similar characteristics. 3. That leg movements did not involve grooming of any body part. 4. That movements were not coincident with forward or backwards locomotion of > 1/2 body length. 5. Where a fly was positioned on the agar/sucrose food or attached to the cotton wool at the opposite length of the tube, movements were also not scored. When a bout of leg twitches occurred, videos were assessed in a frame-by-frame manner (frame rate: 30 Hz) to quantify the number of twitches during the bout. Videos were assessed blind to genotype. Bouts of leg twitches were initially identified by one observer, then independently quantified and confirmed to meet the above criteria by two additional observers. The number and duration of leg twitches in each bout for each fly represents the mean of the values scored by the three observers.

**Adult life span analysis**

110 *slo^E366G/+^* and 100 *slo^loxP/+^* adult male flies were age-matched by emptying parental vials < 12 h prior to the start of the experiment. Males were then housed with an excess number of females for 48 h before the start of the experiment, increasing the probability of mating. *slo^E366G/+^* and *slo^loxP/+^* males were separately placed into standard food vials in groups of 10. Vials were flipped onto fresh food every 2 days, after which the number of dead flies on the former food were counted.

**Morphological analysis of pupae**

Pupae were collected from vials housed at 25ºC, aligned above a ruler, and pictures taken with an iPhone 8 (Apple Inc.) through a stereomicroscope. Images were loaded into FIJI for analysis: width and length were measured in pixels and the results converted to mm in Microsoft Excel (Microsoft Excel for Mac, v 16.22).

**Statistics**

Data populations were initially examined for normal distributions using the Shapiro-Wilk test. Statistical differences in normally distributed populations were tested for via unpaired t-tests with Welch’s correction for non-identical variance, or two-way ANOVA with Sidak’s multiple comparisons test. Non-normal populations were assessed via Mann-Whitney U-test or Kruskal-Wallis test with Dunn’s post-hoc test. Differences in lifespan and the distribution of locomotor speeds were analysed via Kolmogorov-Smirnov test.

**B. Supplemental Figures, Figure legends and Video Legends**

**
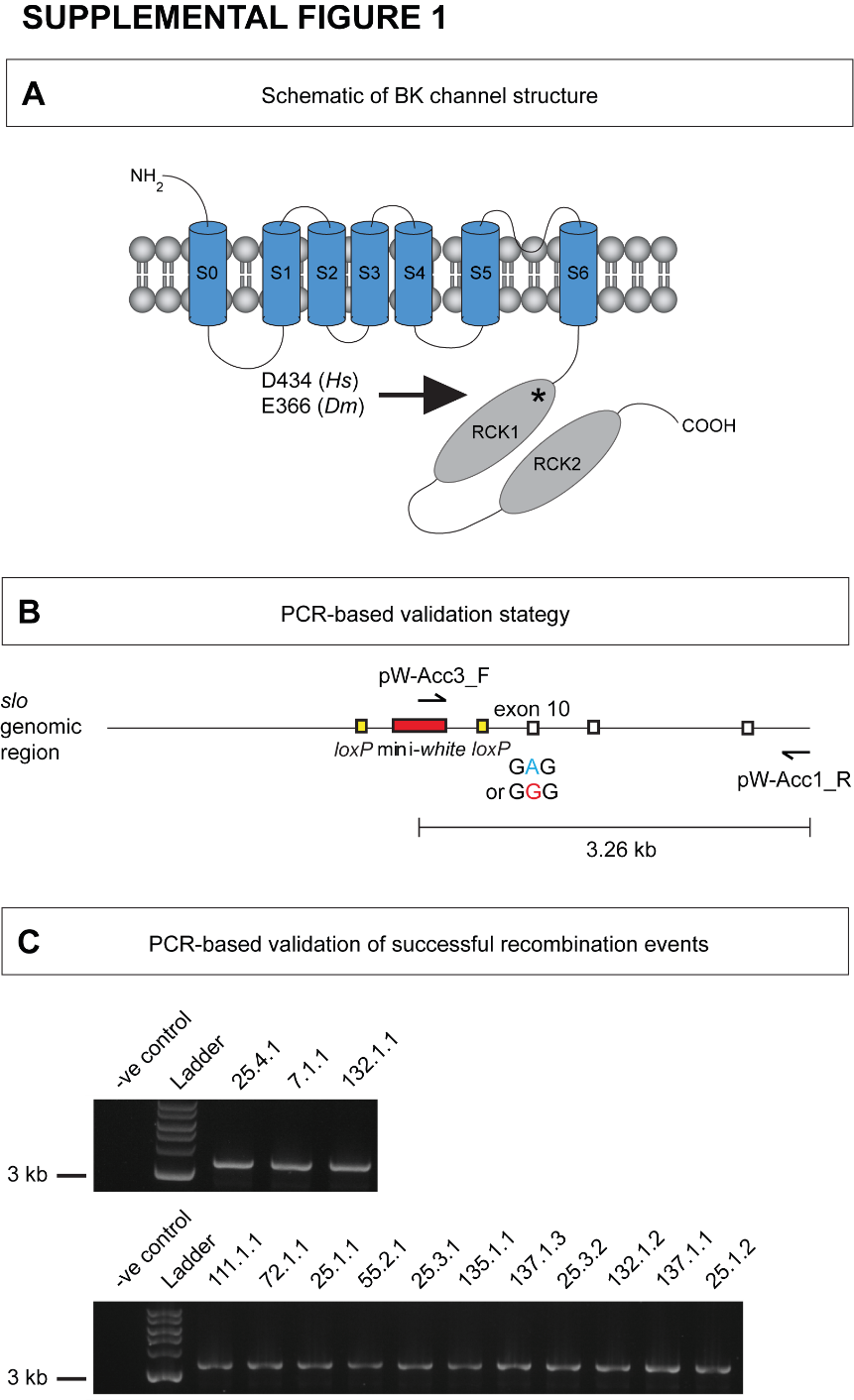
**

**Supplemental Fig. 1.** (A) Schematic showing approximate location of the D434 and E366 residues in the RCK1 domain of human and *Drosophila* hSlo1/SLO. (B) Schematic illustrating the method for validating molecular integration of the *slo*^E366G^ and *slo^loxP^* alleles via ends-out homologous recombination. The region surrounding *slo* exon 10 is shown. Potential recombinants were initially identified by the presence of non-white eye colour due to the mini-*white*^+^ marker, then validated through PCR via the strategy shown in (B). (C) Validated recombinants are denoted by a PCR product of approximately 3.3 kb amplified from single-fly genomic DNA that was absent in non-recombinant control DNA (-ve control).


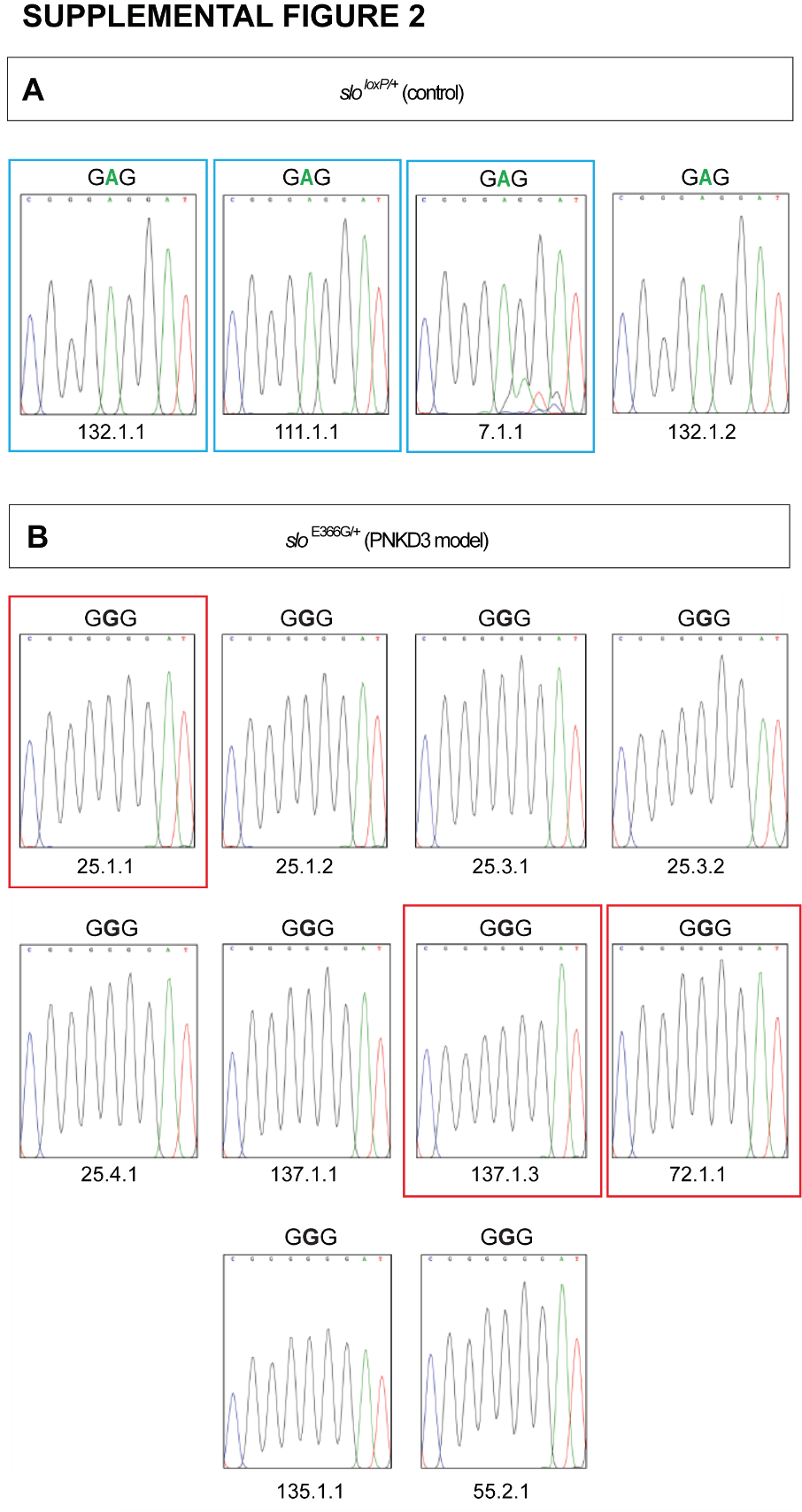


**Supplemental Fig. 2.** Sequence validation of four independent *slo^loxP^* recombinant lines harbouring the genomically encoded glutamic acid (GAG) at position 366 and ten independent *slo*^E366G^ recombinant lines harbouring the artificially introduced glycine (GGG) at the same position. Lines selected for subsequent out-crossing into the iso31 background are noted in blue and red.


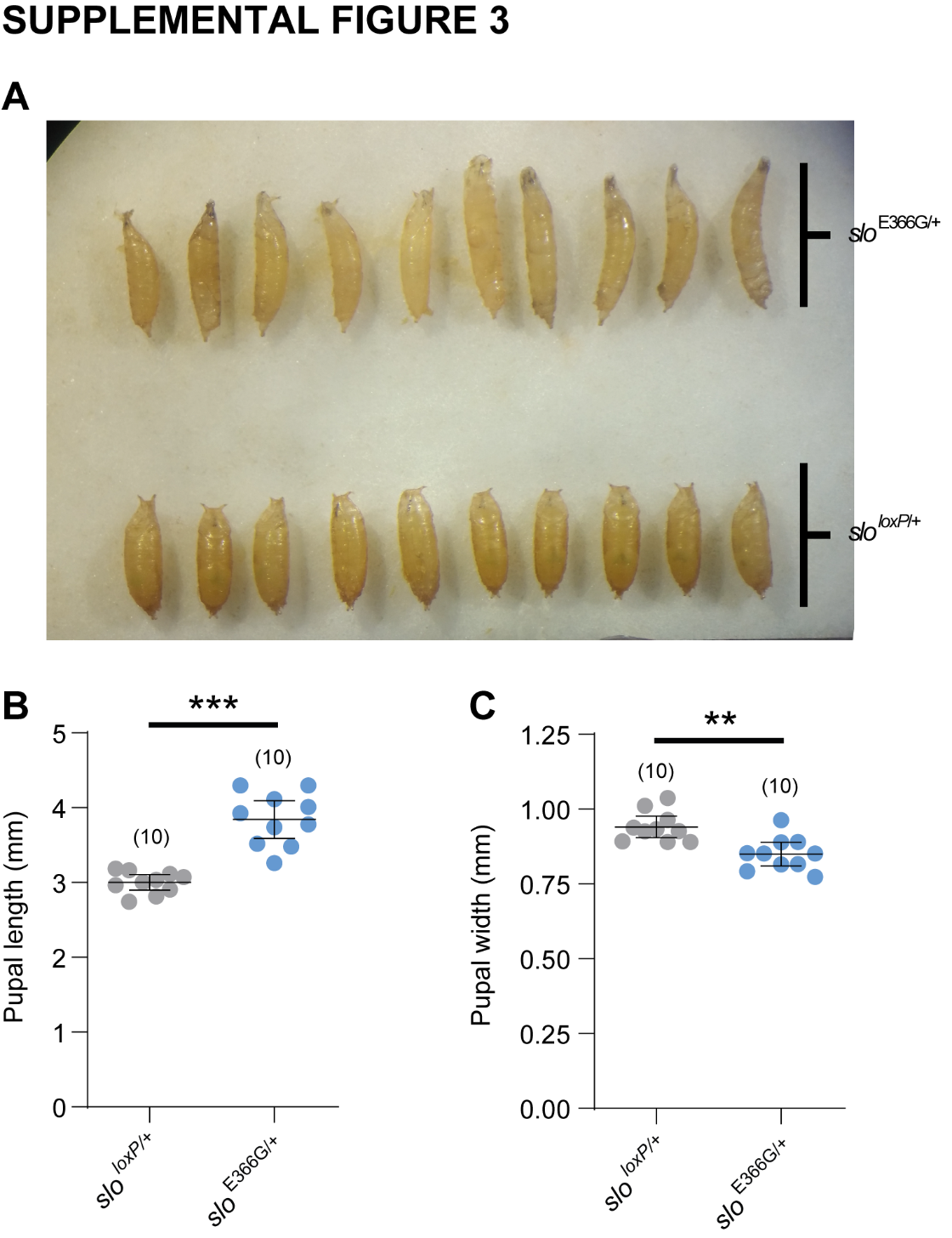


**Supplemental Fig. 3.** Aberrant morphology of *slo*^E366G/E366G^ pupae. (A) Images of *slo*^E366G/E366G^ (top row) and *slo^loxP/loxP^* pupae (bottom row). (B-C) Quantification of pupal dimensions. *slo*^E366G/E366G^ exhibited an increase in length (B) and decrease in width (C) compared to *slo^loxP/loxP^* pupae. Error bars: mean and 95% Confidence Interval (CI). n = 10 for both genotypes. **p<0.005, ***p<0.0005, unpaired t-test with Welch’s correction.

**
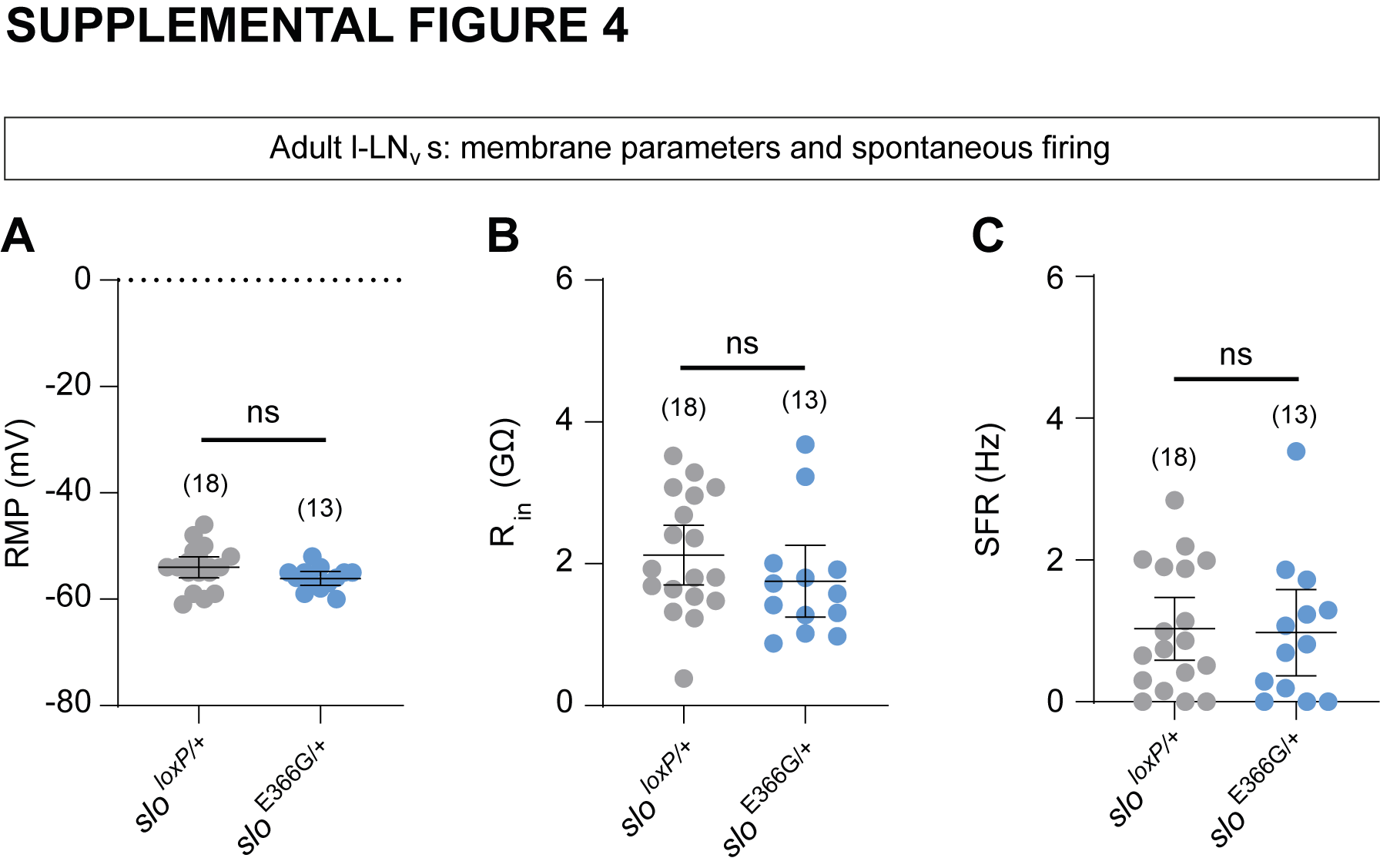
**

**Supplemental Fig. 4.** Resting membrane potential (RMP) (A), membrane resistance (R_in_) (B) and spontaneous firing rate (SFR) (C) in adult male *slo^loxP^*^/+^ or *slo*^E366G/+^ l-LN_v_s recorded at ZT18-20. Each dot represents a recording from an individual neuron; n-values are noted. Recordings were obtained from > 9 flies per genotype. Error bars: mean and 95% CI. ns – p>0.05, unpaired t-test with Welch’s correction (A) or

Mann-Whitney U-test (B, C).

**
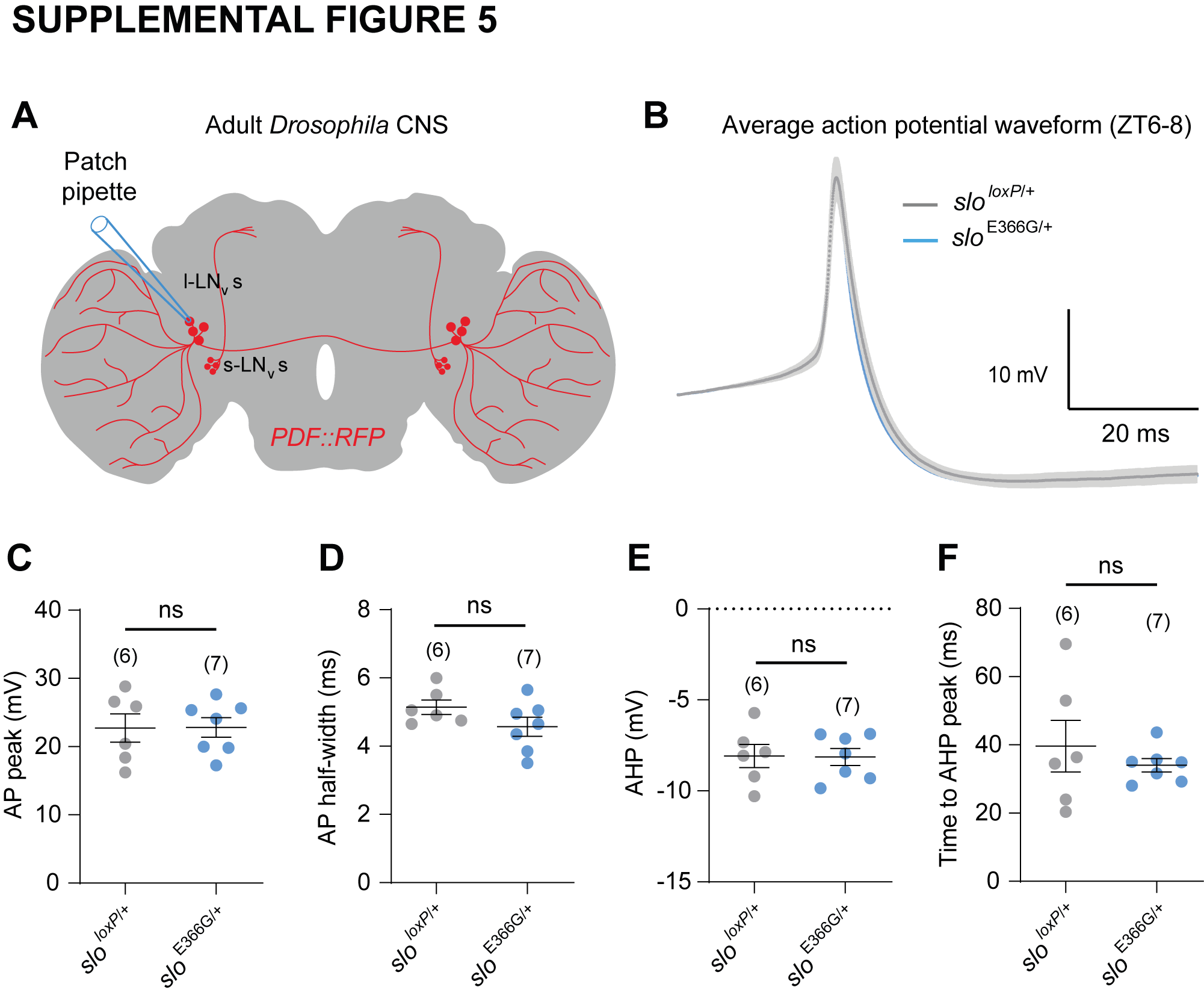
**

**Supplemental Fig. 5**. (A) Illustration showing morphology of l-LN_v_s labelled with *PDF* promoter-driven RFP (*PDF::RFP*) and location of patch-clamp recording sites. (B) Average AP waveforms in l-LN_v_s at ZT6-8 (n = 6-7). Darker and lighter shades show mean and standard error of the mean (SEM). (C-F) l-LN_v_ AP and AHP parameters. n-values are noted. Error bars: 95% CI. ns – p>0.05, unpaired t-test with Welch’s correction.

**
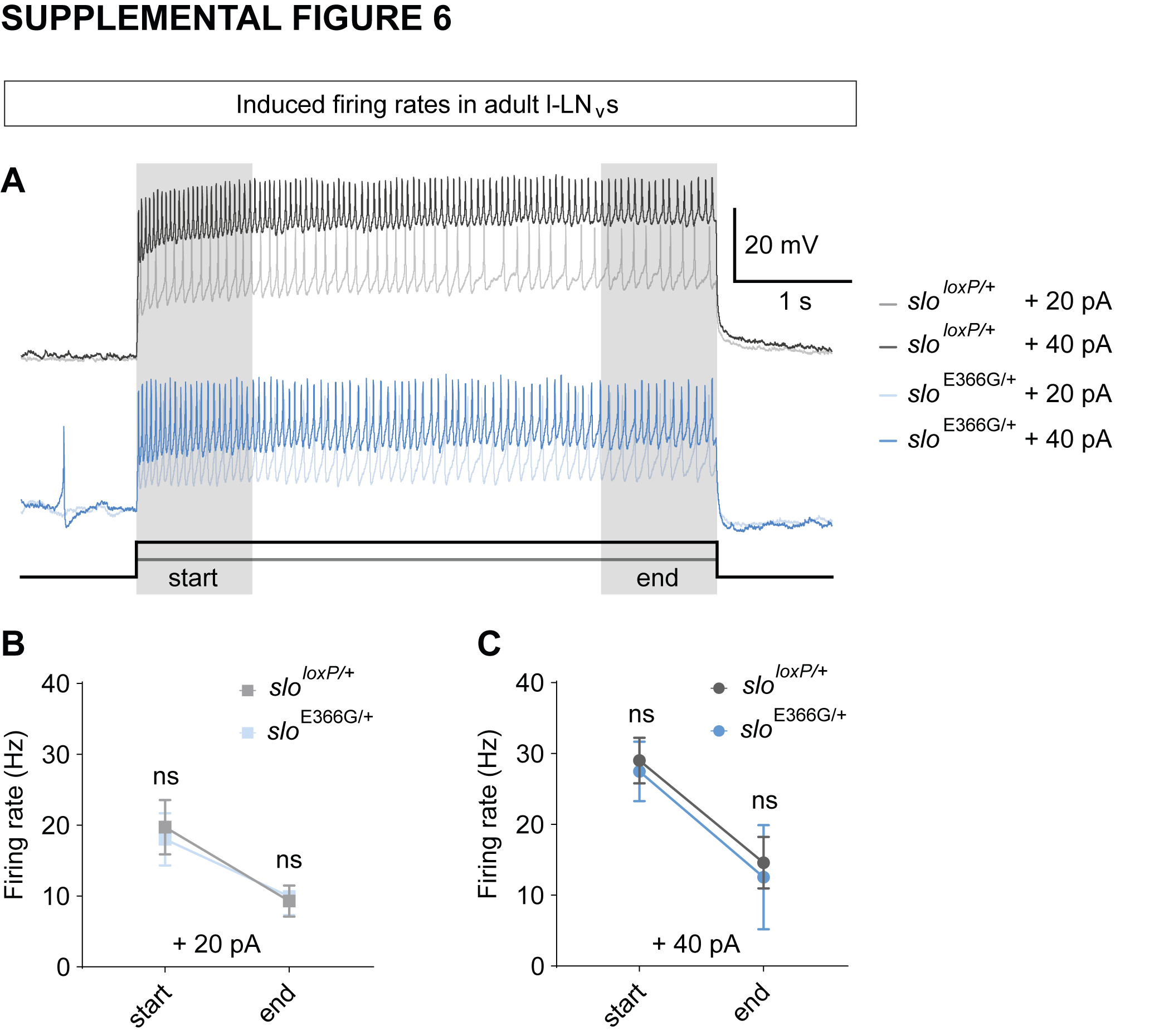
**

**Supplemental Fig. 6.** (A) Representative traces showing AP firing in adult male l-LN_v_s from the *slo^loxP^*^/+^ or *slo*^E366G/+^ backgrounds induced by either +20 pA or +40 pA current injections. (B-C) Mean firing rates in adult male l-LN_v_s from the *slo^loxP^*^/+^ or *slo*^E366G/+^ backgrounds at the start or end of AP trains (as indicated in A) induced by +20 pA or +40 pA current injections. N-values are as follows. +20 pA *slo^loxP^*^/+^: n = 14, +20 pA *slo*^E366G/+^: n = 17, +40 pA *slo^loxP^*^/+^: n = 12, +40 pA *slo*^E366G/+^: n = 15. Error bars: Standard Deviation (SD). ns – p>0.05, Mann-Whitney U-test (+20 pA start, +40 pA start, +40 pA end) or unpaired t-test with Welch’s correction (+20 pA end).

**
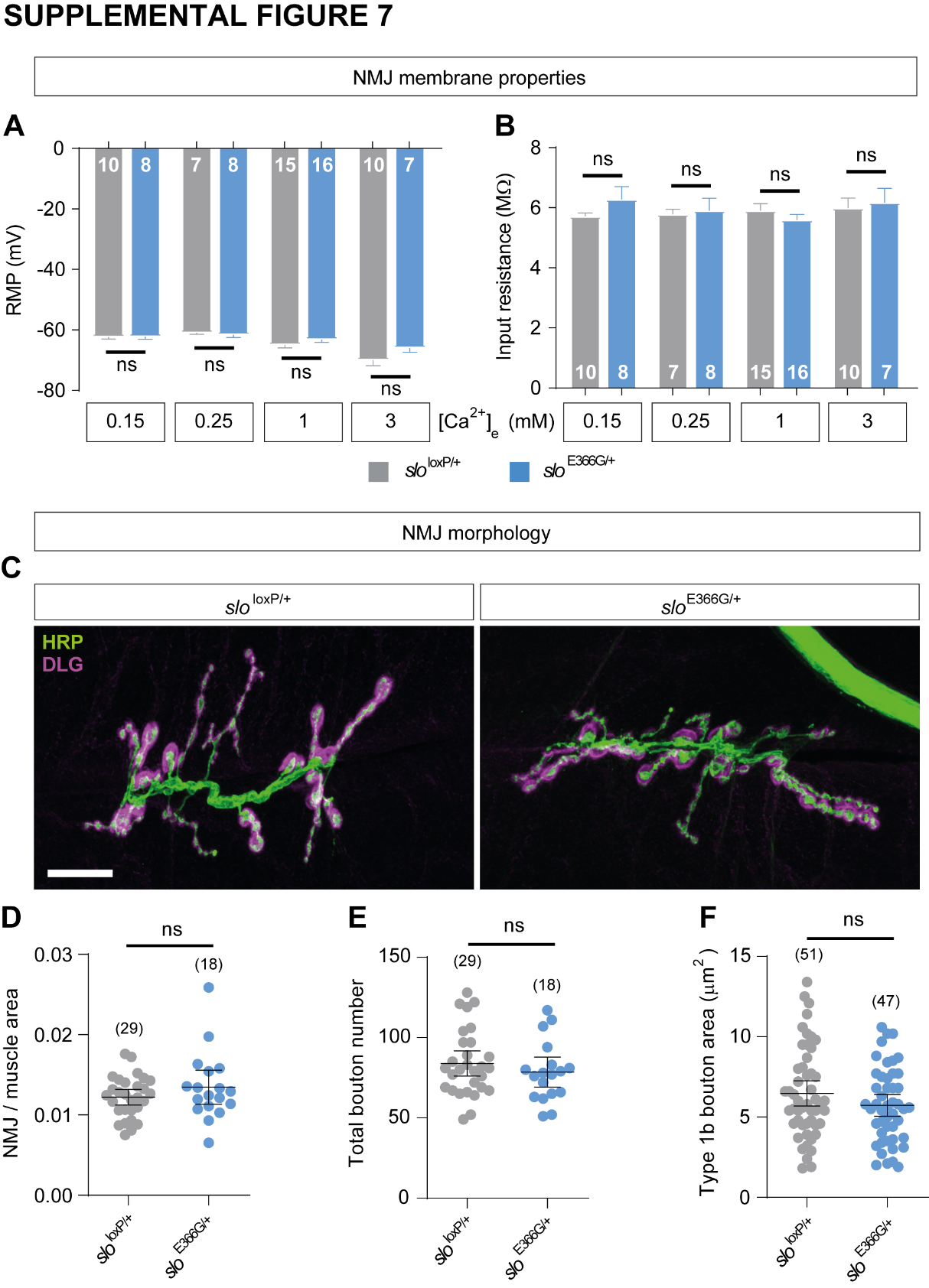
**

**Supplemental Fig. 7.** (A) Resting membrane potential (RMP) of muscle 6 in *slo^loxP^*/+ and slo^E366G/+^ larvae at varying [Ca^2+^]_e_. (B) Muscle input resistance in the same genotypes and [Ca^2+^]_e_. n-values are noted. (C) Representative confocal images of HRP-labelled motoneurons innervating muscle 6/7, segment 3 of the 3^rd^ instar larval body wall. The post-synaptic sub-synaptic reticulum is labelled with anti-Discs Large (DLG). Scale bar: 20 µm. (D) NMJ area of *slo^loxP^*^/+^ and *slo*^E366G/+^ larvae normalized to the corresponding area of muscle 6/7. (E) Total bouton number (type 1s and type 1b) of motoneurons innervating muscle 6/7 in *slo^loxP^*^/+^ and slo^E366G/+^ larvae. (F) Area of type 1b boutons in *slo^loxP^*^/+^ and slo^E366G/+^ larvae. n-values are noted. In D-F, dots represent measurements of NMJ size/bouton number from individual larvae (D, E) or from individual synaptic boutons (E). Error bars: mean and 95% CI. In A-B: ns – p>0.05, Mann-Whitney U-test (A: 0.25 mM and 1 mM Ca^2+^) or unpaired t-test with Welch’s correction (all other comparisons). In D-F: ns – p>0.05, Mann-Whitney U-test (D) or unpaired t-test with Welch’s correction (E, F).

**
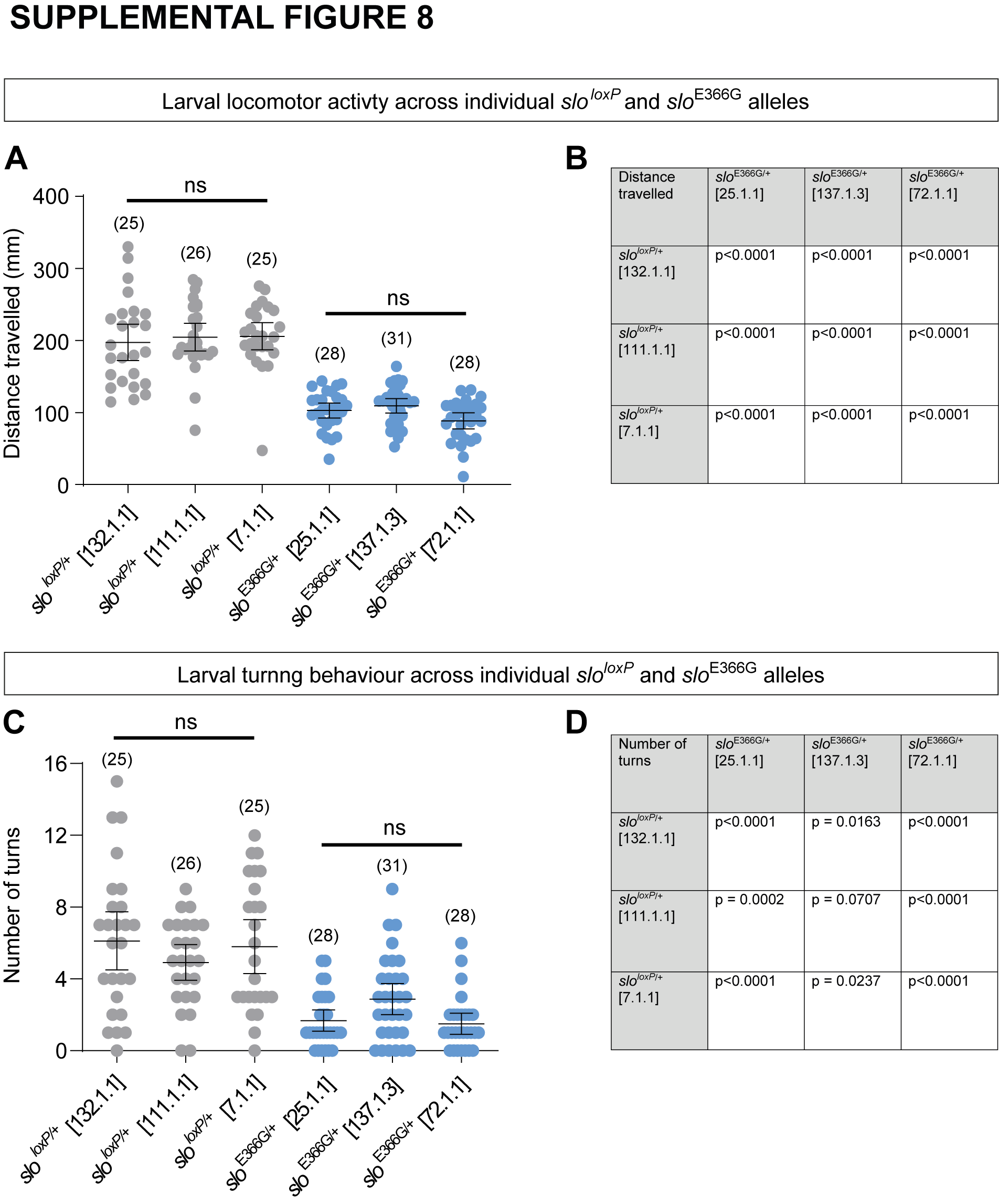
**

**Supplemental Fig. 8.** (A) Mean distance travelled over 1 min by *slo*^E366G/+^ and *slo^loxP^*^/+^ L3 larvae. No significant difference in distance travelled was detected between L3 larvae heterozygous for independently derived insertions of the same *slo* allele (E366G or *loxP*) Kruskal-Wallis test with Dunn’s post-hoc test). (B) Nine pairwise statistical comparisons of distance travelled between L3 larvae heterozygous for three *slo*^E366G/+^ and three *slo^loxP^*^/+^ alleles. Note that all comparisons are highly statistically significant: p<0.0001, Kruskal-Wallis test with Dunn’s post-hoc test. (C) Number of turns initiated over 1 min by *slo*^E366G/+^ and *slo^loxP^*^/+^ L3 larvae. No significant difference in the number of turns initiated over 1 min was detected between L3 larvae heterozygous for independently derived insertions of the same *slo* allele (E366G or *loxP*) (Kruskal-Wallis test with Dunn’s post-hoc test). (D) Nine pairwise statistical comparisons of number of turns between L3 larvae heterozygous for three *slo*^E366G/+^ and three *slo^loxP^*^/+^ alleles. Note that all comparisons are statistically significant (Kruskal-Wallis test with Dunn’s post-hoc test) apart from one comparison which is close to achieving significance (p = 0.0707). n-values are noted. Error bars: mean and 95% CI.

**
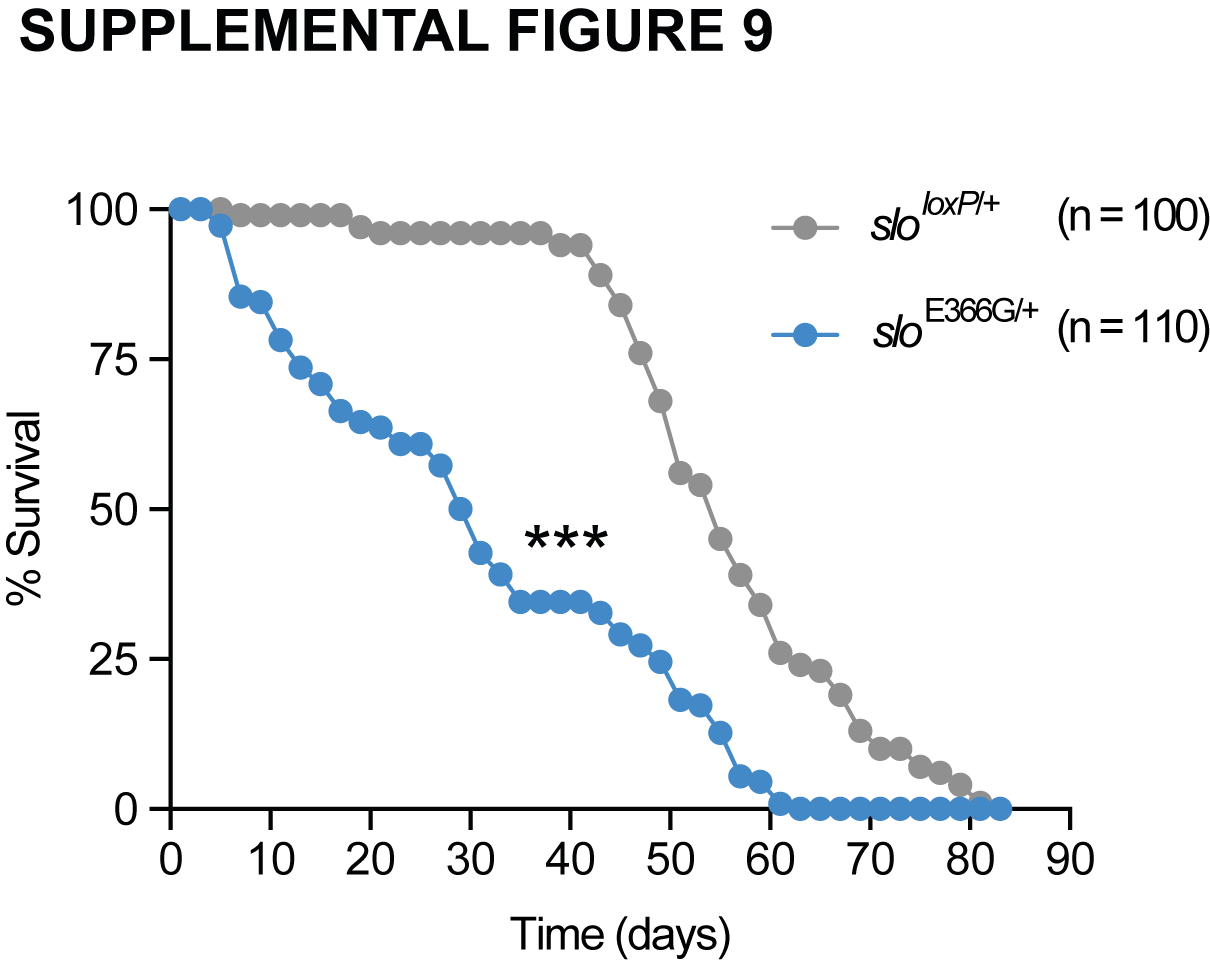
**

**Supplemental Fig. 9.** Mortality rates of *slo^loxP/+^* and *slo*^E366G/+^ adult males. *slo*^E366G^*/+* males show a marked decrease in life span compared to *slo^loxP/+^* males. n-values are shown. ***p<0.0005, Kolmogorov-Smirnov test.

**
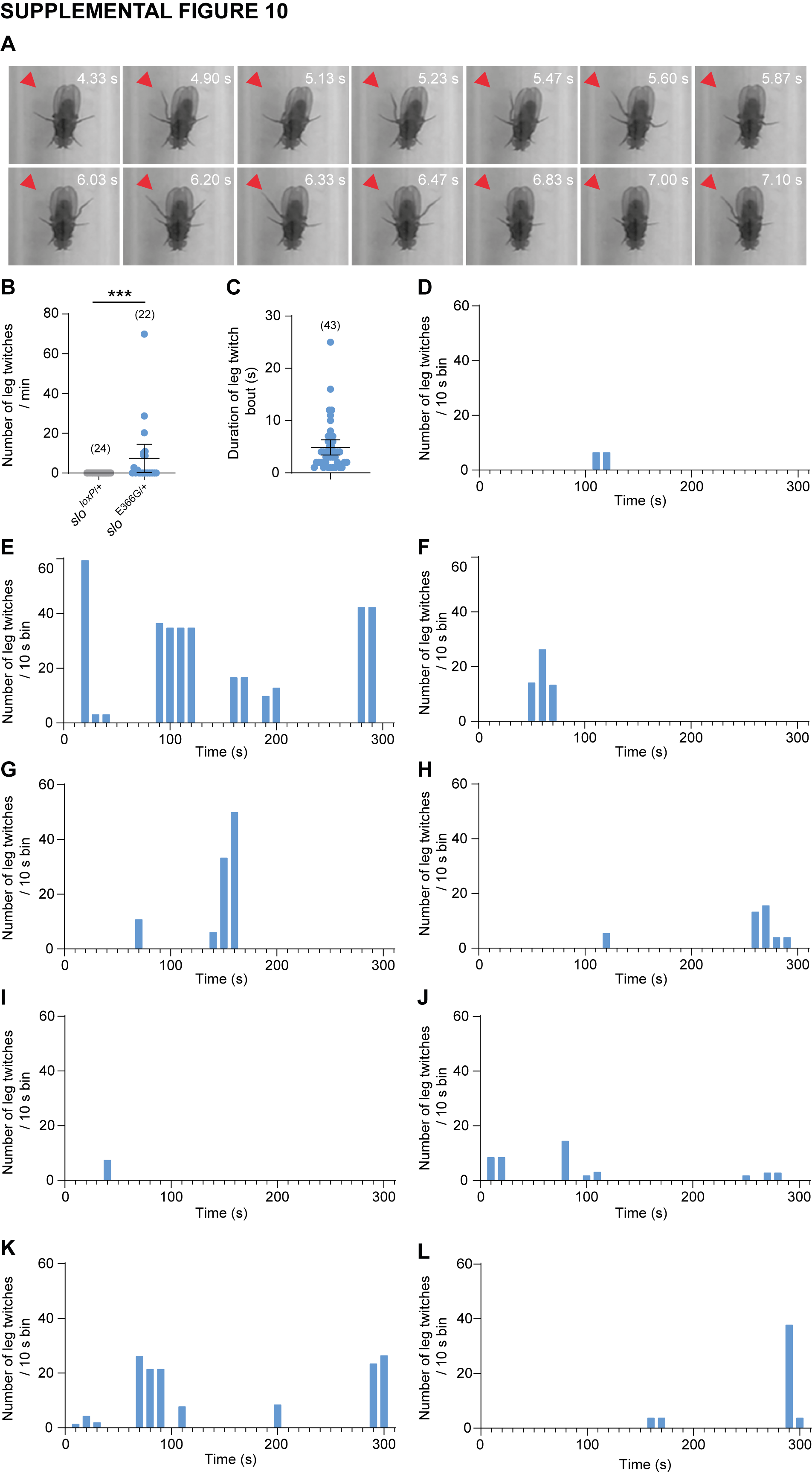
**

**Supplemental Fig. 10.**

(A) Example still images of a single *slo*^E366G/+^ male exhibiting sporadic leg twitches. Arrows point to rapid extensions and contractions of the rear right leg (also see Supplemental Video 2). (B) Frequency of leg twitches in adult male *slo^loxP^*^/+^ and *slo*^E366G/+^ flies. (C) Mean duration of individual bouts of leg twitches in *slo*^E366G/+^ males. n = 43 bouts across 13 flies. (D-L) Occurrence of leg twitch bouts over time in eight additional *slo*^E366G/+^ males over a 5 min period. Each graph represents an individual *slo*^E366G/+^ male. Time is divided into 10 s bins. Where a bout overlapped 10 s bins, the total number of leg twitches was divided evenly between the two bins. Leg twitches were clustered into defined bouts rather than spread uniformly across the 5 min period.


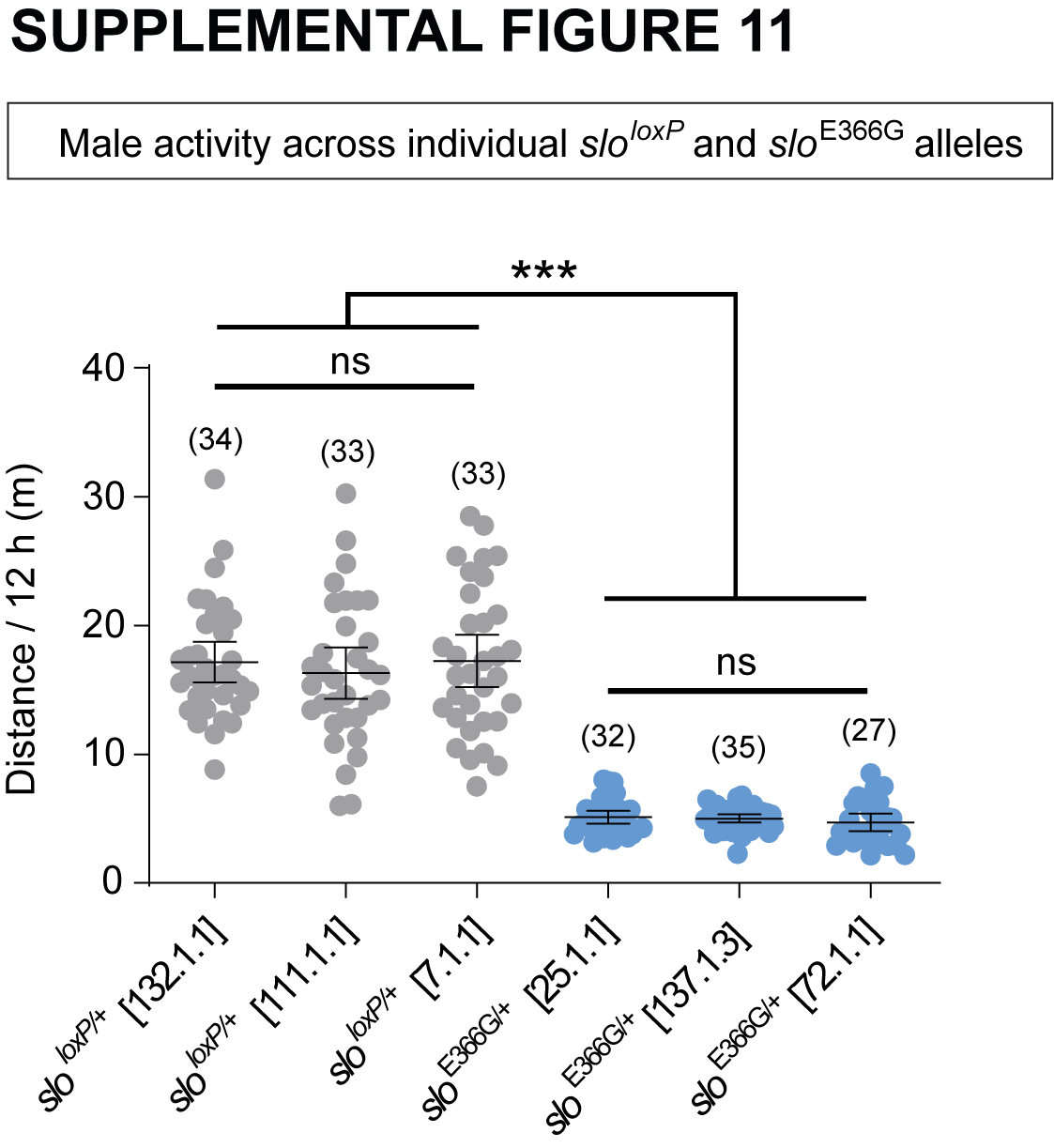


**Supplemental Fig. 11.** Mean distance travelled over 12 h by adult male flies heterozygous for three independently derived *slo*^E366G^ and *slo^loxP^* alleles. n-values are shown. No significant difference in distance travelled was detected between adults heterozygous for independently derived insertions of the same *slo* allele (E366G or *loxP*), whereas all pairwise comparisons of independently derived *slo*^E366G^ and *slo^loxP^* alleles were highly statistically significant. Error bars: mean ± 95% CI. ns – p>0.05, ***p<0.0005, Kruskal-Wallis test with Dunn’s post-hoc test.


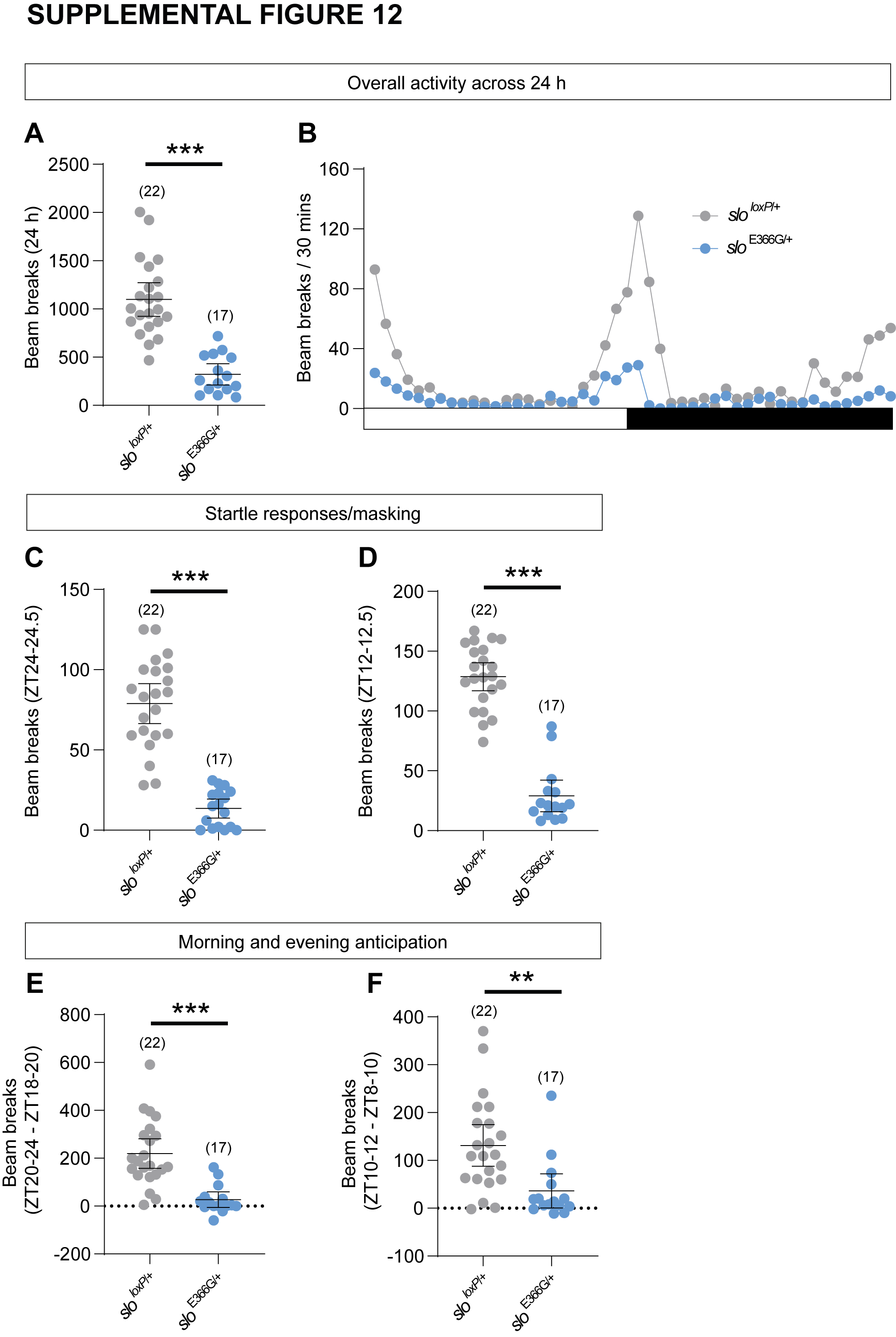


**Supplemental Fig. 12.** (A) Overall activity, represented as number of infra-red beam breaks recorded by the *Drosophila* Activity Monitor (DAM) system, over a 24 h period in 12 h light: 12 h dark conditions, in *slo^loxP^*^/+^ and *slo*^E366G/+^ males. (B) Distribution of beam breaks across the 24 h period, in 30 min intervals. Light bar: lights-on. Black bar: lights-off. Dots represent mean activity during each 30 min window. *slo*^E366G/+^ males clearly exhibited reduced movement across the 24 h period compared to *slo^loxP^*^/+^ males. (C-D) Magnitude of startle responses (also known as masking) in response to lights-on (C) or lights-off (D) in *slo^loxP^*^/+^ and *slo*^E366G/+^ males. Startle responses were defined as the activity during 30 mins following lights-on or lights-off minus the preceding 30 mins activity per fly. (E-F) Magnitude of clock-driven anticipatory increases in activity prior to lights-on (morning anticipation; E) or lights-off (evening anticipation; F) in *slo^loxP^*^/+^ and *slo*^E366G/+^ males. Morning anticipation was defined as the activity during ZT20-24 minus the preceding two hours (E). Evening anticipation was defined as the activity during ZT10-12 minus the preceding two hours (F). n-values are shown. Error bars: mean and 95% CI. **p<0.005, ***p<0.0005, unpaired t-test with Welch’s correction (A) or Mann-Whitney U-test (C-F).


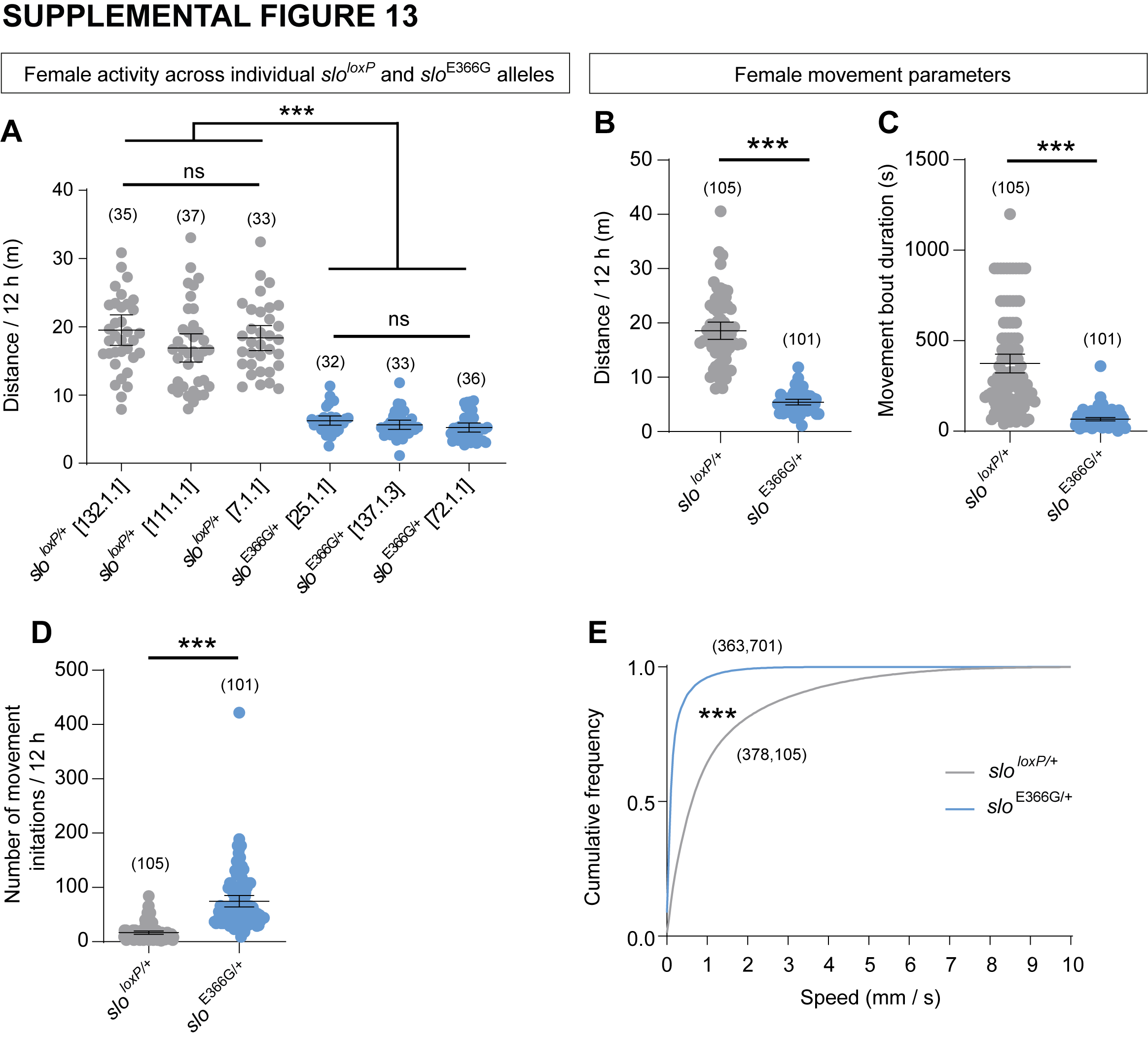


**Supplemental Fig. 13**. (A) Mean distance travelled over 12 h by adult female flies heterozygous for three independently derived *slo*^E366G^ and *slo^loxP^* alleles, measured by the DART system. No significant difference in distance travelled was detected between adults heterozygous for independently derived insertions of the same *slo* allele (E366G or *loxP*), whereas all pairwise comparisons of independently derived *slo*^E366G^ and *slo^loxP^* alleles were highly statistically significant. (B-E) Movement parameters in adult female *slo^loxP^*^/+^ and *slo*^E366G/+^ flies. (B) Total distance travelled by *slo^loxP^*^/+^ and *slo*^E366G/+^ females over 12 h. (C-D) Duration of movement bouts (C) and number of movement initiations (D) between 0-1 h following lights-on in 12 h light: 12 h dark conditions in *slo^loxP^*^/+^ and *slo*^E366G/+^ adult females. (E) Cumulative distribution of speeds during the same 1 h time period in *slo^loxP^*^/+^ and *slo*^E366G/+^ adult females. n-values are noted. Error bars: mean and 95% CI. ns – p>0.05, ***p<0.0005, Mann-Whitney U-test (A-D) or Kolmogorov-Smirnov test (E).

**Supplemental Video 1.** Video shows representative examples of waves of motoneuron excitation in the ex vivo larval ventral nerve cord (VNC) visualised via GCaMP6m driven by the glutamatergic neuron driver *ok371*-Ga4. In the *slo^loxP^*^/+^ control VNC (left), repetitive waves of excitation traveling from posterior to anterior segments can clearly be observed. Unilateral motoneuron excitation in the upper segments can also be infrequently observed. In the *slo*^E366G/+^ VNC (right), both the magnitude and frequency of increases in motoneuron GCaMP6m fluorescence is reduced, indicative of perturbed input from the upstream central pattern generator.

**Supplemental Video 2.** Video shows representative examples of three *slo^loxP^*^/+^ (left) or *slo*^E366G/+^ (right) adult male flies freely moving in glass tubes containing an agar-sucrose food source. Tubes are horizontally placed. Note the more rapid speed and increased continuity of movement of *slo^loxP^*^/+^ compared to *slo*^E366G/+^ males. Subsequent zoom of the left-hand *slo*^E366G/+^ ­male illustrates a bout of unilateral leg-twitches, similar instances of which were frequently observed in *slo*^E366G/+^ but not *slo^loxP^*^/+^ flies.

**C. Supplemental References**

1. Staber CJ, Gell S, Jepson JE, et al. Perturbing A-to-I RNA editing using genetics and homologous recombination. *Methods Mol Biol* 2011;718:41-73. doi: 10.1007/978-1-61779-018-8_3 [published Online First: 2011/03/04]

2. Langley CH, Stevens K, Cardeno C, et al. Genomic variation in natural populations of Drosophila melanogaster. *Genetics* 2012;192(2):533-98. doi: 10.1534/genetics.112.142018 [published Online First: 2012/06/08]

3. Pool JE, Corbett-Detig RB, Sugino RP, et al. Population Genomics of sub-saharan Drosophila melanogaster: African diversity and non-African admixture. *PLoS Genet* 2012;8(12):e1003080. doi: 10.1371/journal.pgen.1003080 [published Online First: 2013/01/04]

4. Love MI, Huber W, Anders S. Moderated estimation of fold change and dispersion for RNA-seq data with DESeq2. *Genome Biol* 2014;15(12):550. doi: 10.1186/s13059-014-0550-8 [published Online First: 2014/12/18]

5. Wu JS, Luo L. A protocol for dissecting Drosophila melanogaster brains for live imaging or immunostaining. *Nat Protoc* 2006;1(4):2110-5. doi: 10.1038/nprot.2006.336 [published Online First: 2007/05/10]

6. Jepson JE, Shahidullah M, Lamaze A, et al. dyschronic, a Drosophila homolog of a deaf-blindness gene, regulates circadian output and Slowpoke channels. *PLoS Genet* 2012;8(4):e1002671. doi: 10.1371/journal.pgen.1002671 [published Online First: 2012/04/26]

7. Buhl E, Bradlaugh A, Ogueta M, et al. Quasimodo mediates daily and acute light effects on Drosophila clock neuron excitability. *Proc Natl Acad Sci U S A* 2016;113(47):13486-91. doi: 10.1073/pnas.1606547113 [published Online First: 2016/11/09]

8. Ruben M, Drapeau MD, Mizrak D, et al. A mechanism for circadian control of pacemaker neuron excitability. *J Biol Rhythms* 2012;27(5):353-64. doi: 10.1177/0748730412455918 [published Online First: 2012/09/27]

9. Tabuchi M, Monaco JD, Duan G, et al. Clock-Generated Temporal Codes Determine Synaptic Plasticity to Control Sleep. *Cell* 2018;175(5):1213-27 e18. doi: 10.1016/j.cell.2018.09.016 [published Online First: 2018/10/16]

10. Imlach WL, Beck ES, Choi BJ, et al. SMN is required for sensory-motor circuit function in Drosophila. *Cell* 2012;151(2):427-39. doi: 10.1016/j.cell.2012.09.011 [published Online First: 2012/10/16]

11. Feng Y, Ueda A, Wu CF. A modified minimal hemolymph-like solution, HL3.1, for physiological recordings at the neuromuscular junctions of normal and mutant Drosophila larvae. *J Neurogenet* 2004;18(2):377-402. doi: 10.1080/01677060490894522 [published Online First: 2005/03/15]

12. McLachlan EM, Martin AR. Non-linear summation of end-plate potentials in the frog and mouse. *J Physiol* 1981;311:307-24. doi: 10.1113/jphysiol.1981.sp013586 [published Online First: 1981/02/01]

13. Klose MK, Dason JS, Atwood HL, et al. Peptide-induced modulation of synaptic transmission and escape response in Drosophila requires two G-protein-coupled receptors. *J Neurosci* 2010;30(44):14724-34. doi: 10.1523/JNEUROSCI.3612-10.2010 [published Online First: 2010/11/05]

14. Streit AK, Fan YN, Masullo L, et al. Calcium Imaging of Neuronal Activity in Drosophila Can Identify Anticonvulsive Compounds. *PLoS One* 2016;11(2):e0148461. doi: 10.1371/journal.pone.0148461 [published Online First: 2016/02/11]

15. Rao SR, Olechnowicz SWZ, Kratschmer P, et al. Small Animal Video Tracking for Activity and Path Analysis Using a Novel Open-Source Multi-Platform Application (AnimApp). *Sci Rep* 2019;9(1):12343. doi: 10.1038/s41598-019-48841-7 [published Online First: 2019/08/28]

16. Faville R, Kottler B, Goodhill GJ, et al. How deeply does your mutant sleep? Probing arousal to better understand sleep defects in Drosophila. *Sci Rep* 2015;5:8454. doi: 10.1038/srep08454 [published Online First: 2015/02/14]

17. Chen KF, Lowe S, Lamaze A, et al. Neurocalcin regulates nighttime sleep and arousal in Drosophila. *Elife* 2019;8 doi: 10.7554/eLife.38114 [published Online First: 2019/03/14]
